# Suppression of *Staphylococcus aureus* biofilm formation and virulence by a benzimidazole derivative, UM-C162

**DOI:** 10.1038/s41598-018-21141-2

**Published:** 2018-02-09

**Authors:** Cin Kong, Chin-Fei Chee, Katharina Richter, Nicky Thomas, Noorsaadah Abd. Rahman, Sheila Nathan

**Affiliations:** 1 School of Biosciences and Biotechnology, Faculty of Science and Technology, Universiti Kebangsaan Malaysia, 43600 UKM Bangi Selangor, Malaysia; 2 Nanotechnology & Catalysis Research Centre, University of Malaya, 50603 Kuala Lumpur, Malaysia; 3 Department of Surgery, Basil Hetzel Institute for Translational Health Research, The University of Adelaide, Adelaide, South Australia Australia; 4 Adelaide Biofilm Test Facility, Sansom Institute for Health Research, University of South Australia, Adelaide, South Australia Australia; 5 School of Pharmacy and Medical Sciences, University of South Australia, Adelaide, South Australia Australia; 6 Department of Chemistry, Faculty of Science, University of Malaya, 50603 Kuala Lumpur, Malaysia; 7 Present Address: Department of Biomedical Sciences, Faculty of Science, University of Nottingham Malaysia Campus, 43500 Semenyih, Selangor Malaysia

## Abstract

*Staphylococcus aureus* is a major cause of nosocomial infections and secretes a diverse spectrum of virulence determinants as well as forms biofilm. The emergence of antibiotic-resistant *S*. *aureus* highlights the need for alternative forms of therapeutics other than conventional antibiotics. One route to meet this need is screening small molecule derivatives for potential anti-infective activity. Using a previously optimized *C*. *elegans* – *S*. *aureus* small molecule screen, we identified a benzimidazole derivative, UM-C162, which rescued nematodes from a *S*. *aureus* infection. UM-C162 prevented the formation of biofilm in a dose-dependent manner without interfering with bacterial viability. To examine the effect of UM-C162 on the expression of *S*. *aureus* virulence genes, a genome-wide transcriptome analysis was performed on UM-C162-treated pathogen. Our data indicated that the genes associated with biofilm formation, particularly those involved in bacterial attachment, were suppressed in UM-C162-treated bacteria. Additionally, a set of genes encoding vital *S*. *aureus* virulence factors were also down-regulated in the presence of UM-C162. Further biochemical analysis validated that UM-C162-mediated disruption of *S*. *aureus* hemolysins, proteases and clumping factors production. Collectively, our findings propose that UM-C162 is a promising compound that can be further developed as an anti-virulence agent to control *S*. *aureus* infections.

## Introduction

*Staphylococcus aureus* is a clinical pathogen that causes both human and animal infections, ranging from mild superficial infections to toxin-associated diseases and severe life-threatening invasive infections^1^. *S*. *aureus* is acknowledged as a key agent implicated in nosocomial infections resulting in significant morbidity and mortality among hospitalized patients. This is in part due to its ability to adhere to the surface of indwelling medical devices and develop biofilm, a multilayered structure comprising of bacterial communities embedded within the extracellular hydrated polymeric matrix^2^.

To survive the onslaught of the host immune response and establish an infection, *S*. *aureus* expresses a comprehensive array of virulence determinants, including extracellular toxins (e.g. hemolysins, leukotoxins and enterotoxins), enzymes (e.g. proteases and coagulases) and surface proteins (e.g. clumping factors, adhesins and *S*. *aureus* surface proteins)^3^. The secretion of these virulence factors and formation of biofilm are controlled by a sophisticated network of virulence regulators such as the *agr*, *sarA*, *saeR/S* and *arlRS*^4^. The diversity of *S*. *aureus* virulence factors along with the ability to form disease-associated biofilm presents a challenge to the host immune defence to fully eradicate the bacteria whilst rendering the bacteria highly tolerant to antibiotics, leading to persistent chronic infections^2^.

The growing frequency of antibiotic-resistant *S*. *aureus* strains as exemplified by methicillin-resistant *S*. *aureus* (MRSA) is a serious threat to the community and highlights a pressing need for alternative therapies that impose minimum selection pressure upon the bacteria. The cumulative understanding of bacterial virulence and pathogenesis opens up new perspectives for developing novel therapies by attenuating bacterial virulence and/or biofilm as alternative treatment options for bacterial infections^5–8^. Recent studies have focused on development of small molecules and phytochemicals to inhibit the formation of biofilm and/or other virulence factors in *S*. *aureus*^9–12^. As most virulence traits are non-essential for bacterial survival, these strategies hold great promise as effective means to control bacterial infections with less probability for selection of resistant populations^13,14^.

Benzimidazole is structurally analogous to naturally occurring nucleotides, making it a key intermediate in the synthesis of new chemical entities of biological interest. The benzimidazole class of compounds are known to possess an extensive range of pharmacological activities such as anti-malarial, anti-cancer and antioxidative effects^15^. In light of the notable therapeutic properties of the benzimidazole scaffold, we performed an *in vivo* screen on a collection of 35 benzimidazole derivatives for potential anti-infective effects using a previously established *C*. *elegans* – *S*. *aureus* anti-infective screen^16^. In an anti-biofilm assay, several compounds reduced the formation of biofilm without inhibiting the growth of *S*. *aureus* and the most potent derivative, 2-(4′-((3-acetamidobenzyl)oxy)-[1,1′-biphenyl]-4-yl)-6-methyl-1*H*-benzimidazole-4-carboxylic acid (referred to as UM-C162 henceforth) was selected for further analysis. Transcriptome profiling was also performed on UM-C162-treated *S*. *aureus* to identify the bacterial virulence factors targeted by UM-C162.

## Results

### Benzimidazole derivatives exhibit anti-infective and anti-biofilm activities

**Figure 1:**
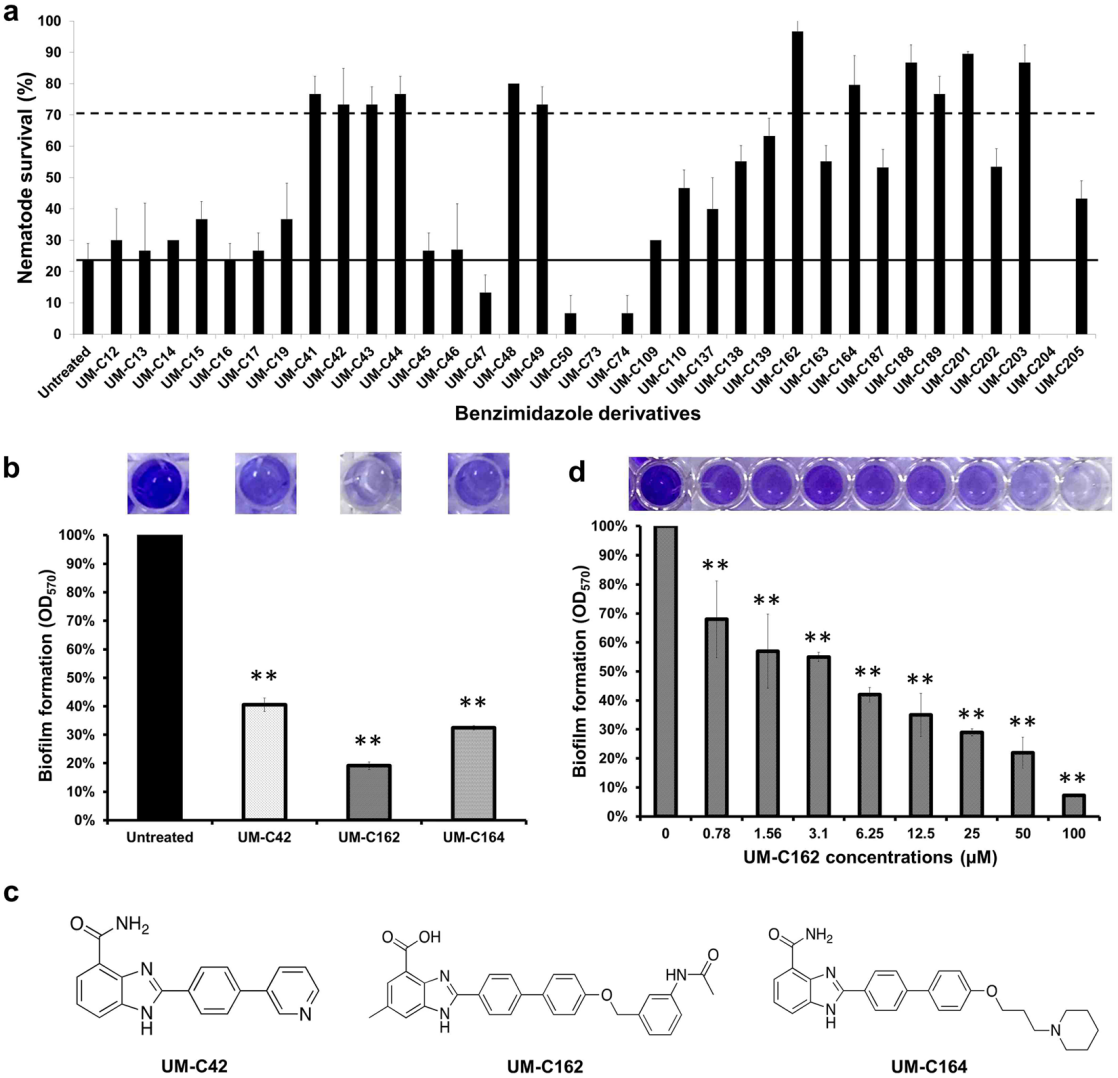
Benzimidazole derivatives rescued *C*. *elegans* from a *S*. *aureus* infection and altered *S*. *aureus* biofilm formation. (**a**) Survival of *S*. *aureus*-infected nematodes upon treatment with individual benzimidazole compounds (100 µM). Results are shown as mean ± SD from a single replicate of two independent screens. The straight line shows the survival of untreated worms while the dashed line demarcates the positive hits. Compounds that promoted the survival of infected worms to > 70% were considered as positive in the screen. (**b**) Effect of benzimidazole compounds (100 µM) towards *S*. *aureus* biofilm formation. (**c**) Molecular structures of compounds UM-C42, UM-C162 and UM-C164 that demonstrated significant anti-biofilm activity. (**d**) Dose-dependent anti-biofilm effect of compound UM-C162. The graphs in (**b**) and (**d**) depict the percentage of biofilm formation after 24 hours incubation of *S*. *aureus* in the presence and absence of compounds. Three independent experiments were conducted. Error bars indicate SEM. Images of representative wells from the crystal-violet biofilm assay are shown. (**) denotes significant difference between untreated and compound-treated bacteria (*p* < 0.01).

In the current study, we extended the utility of a previously optimized *C*. *elegans* – *S*. *aureus* anti-infective screen^16^ to explore the potential anti-infective property of a series of benzimidazole compounds. The premise of the screen was that the addition of individual compounds that are able to rescue the nematodes from a lethal *S*. *aureus* infection are deemed to exhibit potential anti-infective effects. We screened 35 benzimidazole derivatives (Table S1) and observed that 12 compounds increased the survival of infected worms to > 70% at a point when ~80% of the untreated worms succumbed to the infection (Fig. 1a). The 12 positive hits are compounds UM-C41, UM-C42, UM-C43, UM-C44, UM-C48, UM-C49, UM-C162, UM-C164, UM-C188, UM-C189, UM-C201 and UM-C203. All positive hits (at 100 µM) enhanced the survival of *S*. *aureus* infected-worms by 3 to 4-fold relative to the untreated control after 96 hours post-infection. On the other hand, we also noticed a small number of compounds (namely UM-C50, UM-C73, UM-C74 and UM-C204) that accelerated the killing of *S*. *aureus*-infected worms, resulting in a lower survival of nematodes (<10%) as compared to the untreated infected worms (~20%) (Fig. 1a). To address the possible toxic effect of these four compounds on the host, we repeated the assay on worms exposed to heat-killed *Escherichia coli* OP50 in the presence of these compounds. As expected, the survival of the worms decreased significantly as early as 48 hours post-exposure (Fig. S1), indicating that these compounds are indeed toxic to the host.

Previously, Begun *et al*. reported that *S*. *aureus* that overexpressed the *icaADBC* locus and constitutively produced excess biofilm exopolysaccharide, killed worms significantly faster as compared to a strain that produced less biofilm, suggesting that staphylococcal biofilm is a virulence mechanism employed by the bacteria that contributes to death of the infected host^17^. In another study, a benzimidazole-derived molecule (ABC-1) was identified as a broad-spectrum biofilm inhibitor towards both Gram-positive and Gram-negative bacteria^18^. ABC-1 suppresses *S*. *aureus* protein A (SpA) expression and prevents the accumulation of polysaccharide intercellular adhesin (PIA)^19^. Therefore, it is possible that some of the hits identified from the *C*. *elegans* anti-infective screen may regulate the production of biofilm by *S*. *aureus*, thereby extending the survival of infected nematodes. To examine the potential anti-biofilm property of the hits, bacteria were cultured in the presence of the individual compounds and biofilm formation was screened using the 96-well crystal violet biofilm assay^20^. Following 24 hours incubation under static conditions, three of the 12 hits (UM-C42, UM-C162 and UM-C164) dramatically inhibited *S*. *aureus* biofilm development by > 70% (*p* < 0.01). The most pronounced anti-biofilm activity was observed for UM-C162 (Fig. 1b). UM-C42, UM-C162 and UM-C164 at a concentration of 100 µM were able to inhibit biofilm formation to 41 ± 2.4%, 19 ± 1.4% and 32 ± 0.8% of the untreated bacteria, respectively. The molecular structures of these compounds are presented in Fig. 1c. The ability of UM-C42, UM-C162 and UM-C164 to diminish the biofilm-forming capability of *S*. *aureus* suggests that these compounds rescued the nematodes from infection via attenuation of this bacterial virulence factor. As compound UM-C162 was most effective in preventing *S*. *aureus* biofilm formation, we further assessed the effect of this compound on *S*. *aureus* biofilm production across a series of lower concentrations. The results showed that UM-C162 reduced *S*. *aureus* biofilm formation in a concentration-dependent manner after treatment for 24 hours, where supplementation of UM-C162 from 0.78 µM to 100 µM was able to reduce biofilm formation by 8.9% to 68% (Fig. 1d). Based on these data, inhibition of >50% biofilm formation was achieved with 6.25 µM of UM-C162. We thus selected 6.25 µM as the lowest working concentration for the rest of the assays.

### UM-C162 disrupts biofilm formation without affecting *S*. *aureus* growth

**Figure 2:**
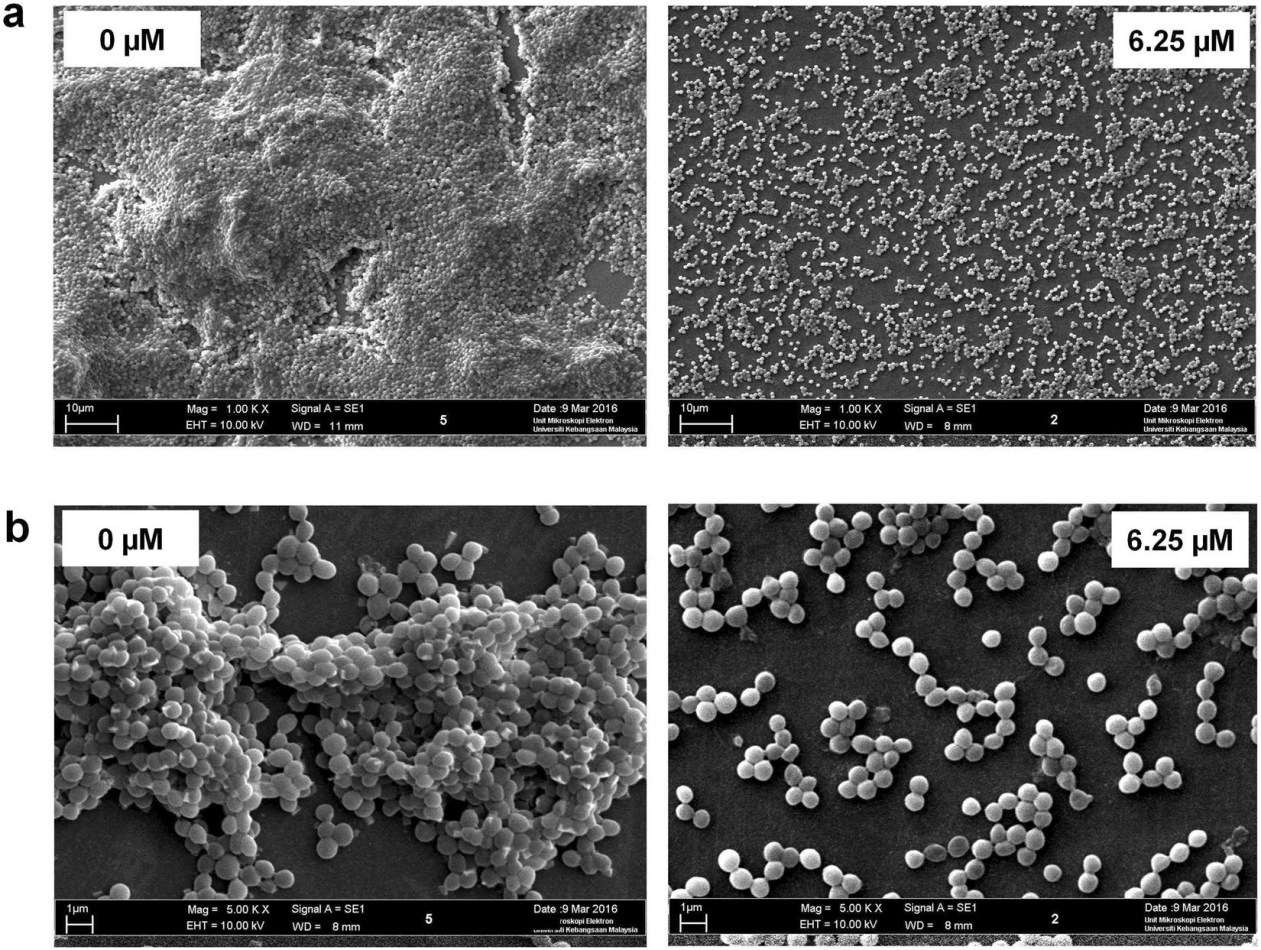
Scanning electron microscopy micrographs of the *S*. *aureus* biofilm structure. (**a**) Biofilm formation by untreated (0 µM) and UM-C162-treated *S*. *aureus* (6.25 µM) following 24 hours incubation (1000x magnification). (**b**) Bacterial cells cluster in untreated *S*. *aureus* (0 µM) and the absence of cell aggregates in UM-C162-treated bacteria (6.25 µM) under 5000x magnification.

To further characterize the effect of UM-C162 on *S*. *aureus* biofilm, *S*. *aureus* was supplemented with 6.25 µM, 12.5 µM and 25 µM UM-C162 and biofilm was allowed to develop on the surface of glass slides in a multi-well plate. The morphology of UM-C162-treated and untreated *S*. *aureus* biofilm architecture formed on the glass slides following 24 hours incubation was visualized using scanning electron microscopy (SEM). Under 1000x magnification, SEM analysis revealed a thick, dense and fully established biofilm consisting of multi-layered bacterial cells (Fig. 2a). Upon treatment with UM-C162, biofilm production was disrupted and a negligible biofilm mass was detected on the glass slide. The bacteria appeared as a monolayer of dispersed cells scattered on the surface (Fig. 2a). At higher magnification (5000x), untreated *S*. *aureus* appeared as large aggregates of cells in overlapped layers (Fig. 2b). In contrast, bacteria treated with 6.25 µM of UM-C162 presented as a uniform layer of cells with negligible clumping (Fig. 2b). A similar observation was noted upon supplementation with higher concentrations of UM-C162 (Fig. S2). These images validate the data from the crystal-violet biofilm assay.

**Figure 3:**
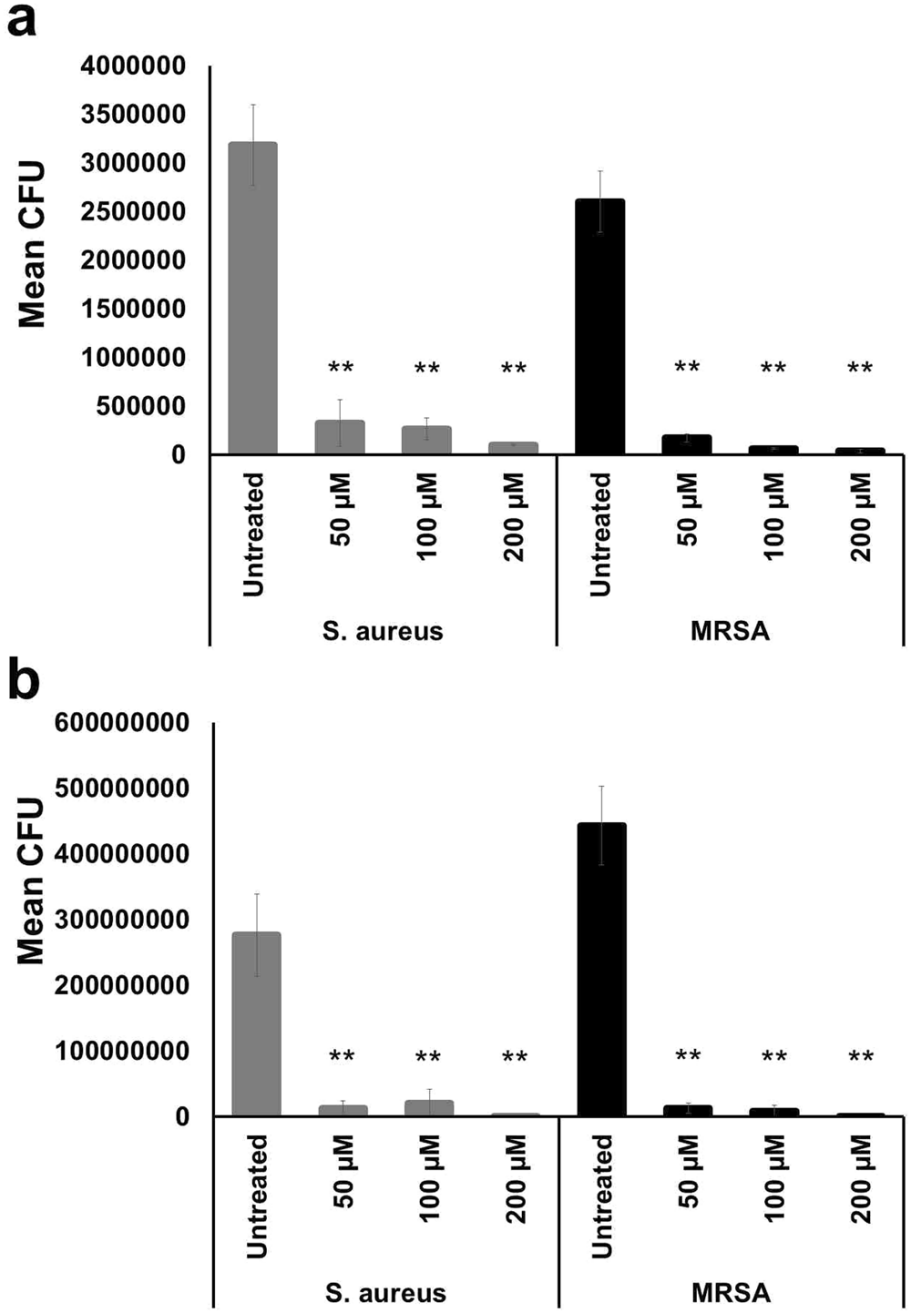
UM-C162 displays anti-biofilm activity in an artificial dermis wound model. Number of *S*. *aureus* and MRSA CFU recovered from biofilm formed on artificial dermis after exposure to various concentrations of UM-C162 for 24 hours in (**a**) biofilm inhibition and (**b**) biofilm eradication assays. Data represent the mean ± SD of two biological replicates. (**) denotes significant difference between untreated and compound-treated bacteria (*p* < 0.001).

We further evaluated the biofilm inhibitory effect and biofilm eradication activity of UM-C162 against two different strains of *S*. *aureus* (*S*. *aureus* ATCC25923 and a MRSA clinical strain) using an artificial dermis wound model^21,22^. To assess the biofilm inhibitory activity, the dermis was supplemented with UM-C162 before the formation of biofilm. On the other hand, in the biofilm eradication assay, we exposed the 24-hour old biofilm to UM-C162 for 24 hours before harvesting the biofilm cells to enumerate bacterial colony forming units (CFU). In the inhibition assay, formation of biofilm was noted in the untreated infected wound whilst no visually detectable biofilm was observed in artificial dermis wounds treated with 50, 100 and 200 µM of UM-C162 for both *S*. *aureus* (Fig. S3a) and MRSA (Fig. S3b). This concentration range was selected based on a preliminary screen across a series of concentrations using the similar artificial dermis model. For the inhibition assay, in the absence of UM-C162, the bacterial load was 3.2 × 10^6^ CFU for *S*. *aureus* and 2.6 × 10^6^ for MRSA. Upon treatment with UM-C162, we observed a significant reduction (*p* < 0.001) in the number of *S*. *aureus* harvested from the artificial dermis i.e. 3.2 × 10^5^ (50 µM), 2.7 × 10^5^ (100 µM) and 1.0 × 10^5^ (200 µM) whilst for MRSA, the number of bacteria was 1.7 × 10^5^ (50 µM), 6.5 × 10^4^ (100 µM) and 4.0 × 10^4^ (200 µM) (Fig. 3a). Similarly, in the eradication assay, UM-C162-treated wounds demonstrated a substantial reduction in bacterial loads for *S*. *aureus* and MRSA (*p* < 0.001) (Fig. 3b). Bacterial loads recovered from the untreated 24-hour old biofilm were 2.7 × 10^8^ CFU for *S*. *aureus* and 4.4 × 10^8^ for MRSA. Bacterial CFU were 1.3 × 10^7^, 2.1 × 10^7^, 1.4 × 10^6^ for *S*. *aureus* and 1.3 × 10^7^, 8.6 × 10^6^ and 3.2 × 10^5^ for MRSA after exposure to UM-C162 at 50, 100 and 200 µM respectively, suggesting that UM-C162 might be able to eradicate the pre-formed biofilms or prevent further biofilm formation in an infected wound.

**Figure 4:**
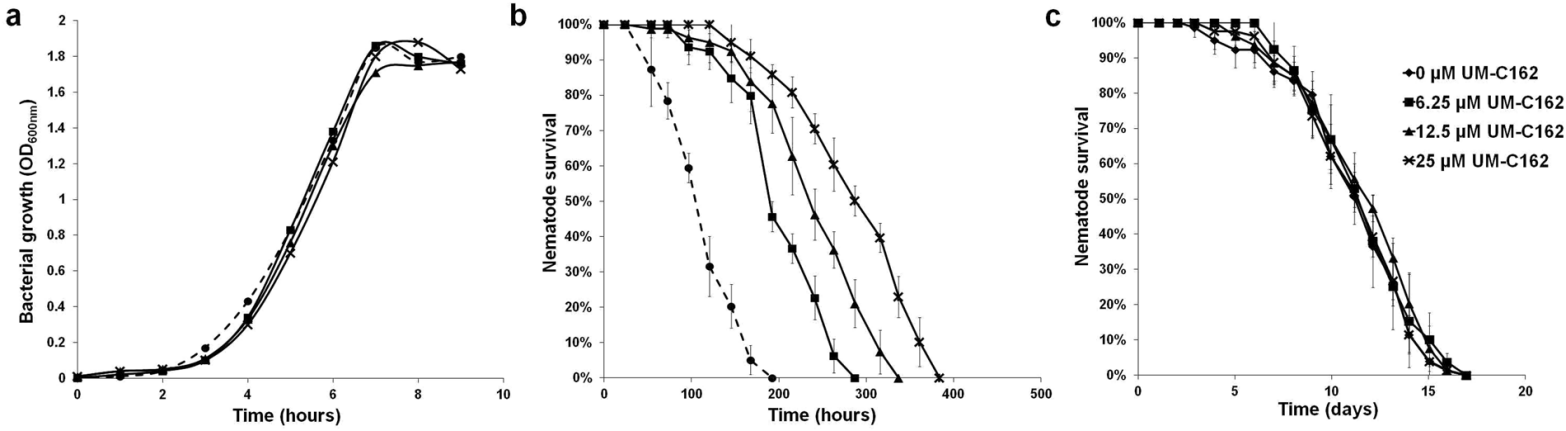
UM-C162 improves the lifespan of *S*. *aureus*-infected nematodes without affecting bacterial growth. (**a**) UM-C162 does not inhibit the growth of *S*. *aureus* in the presence of 6.25 µM, 12.5 µM and 25 µM UM-C162. (**b**) Survival curves of *S*. *aureus*-infected *C*. *elegans* treated with 6.25 µM, 12.5 µM and 25 µM of UM-C162 compared to untreated control (0 µM). UM-C162 at all concentrations tested confers significant enhanced survival in infected nematodes (*p* < 0.0001). (**c**) UM-C162 does not alter the basal lifespan of worms fed on heat killed *E*. *coli* OP50. Graphs in (**b**) and (**c**) show the mean ± SD of six technical replicates (20 worms/replicate) from a representative of two independent experiments.

*S*. *aureus* susceptibility towards UM-C162 was tested across a range of concentrations using the broth microdilution MIC test. Gentamicin was tested in parallel as the positive control and was antimicrobial towards *S*. *aureus* with an MIC of < 30 µg/mL (Fig. S4). On the other hand, *S*. *aureus* supplemented with different concentrations of UM-C162 appeared turbid after an overnight incubation, indicating that over a dose of 0.05 µM to 200 µM, UM-C162 did not inhibit *S*. *aureus* growth (Fig. S4). To confirm the MIC data, the effect of UM-C162 on *S*. *aureus* growth was evaluated by monitoring the absorbance at OD_600nm_ of batch cultures. *S*. *aureus* showed a similar growth rate in the presence and absence of UM-C162 (Fig. 4a), confirming that UM-C162 at concentrations ranging from 6.25 µM to 25 µM did not interfere with *S*. *aureus* growth. These findings also propose that the attenuation of *S*. *aureus* virulence by UM-C162 is mainly due to the compound’s ability to retard biofilm production and not to its antimicrobial activity against planktonic cells.

To further assess the protective role of UM-C162 towards the host *in vivo*, the lifespan of infected nematodes in the presence and absence of UM-C162 was monitored in a survival assay with additional time points and a greater number of worms. *S*. *aureus* killed the nematodes with a mean-time-to-death (TD_mean_) of 114.5 ± 3.7 hours (4.7 days) (Fig. 4b). Complete killing of all nematodes was achieved in 7–8 days. Upon treatment with 6.25 µM, 12.5 µM and 25 µM of UM-C162, the TD_mean_ of *S*. *aureus*-infected worms was markedly improved to 207.1 ± 5.5, 244.5 ± 7.0 and 290.2 ± 7.8 hours, respectively (*p* < 0.0001) (Fig. 4b). Hence, the killing kinetics of infected worms was significantly delayed in a concentration-dependent manner for worms treated with UM-C162.

We then asked if UM-C162 is toxic towards a multicellular eukaryotic organism. To test this, we exposed the worms to various concentrations of UM-C162 in the absence of infection and lifespan of the worms was monitored. No significant difference was observed between the lifespan of UM-C162-treated and untreated worms, where both survived for ~17 days at 25 °C (Fig. 4c), ruling out the possibility that the compound could cause adverse effects onto the host. Moreover, the compound did not further extend the basal lifespan of nematodes, implying that the increased survival of nematodes infected by *S*. *aureus* is unlikely to be an outcome of any lifespan extending property of UM-C162.

### *S*. *aureus* global transcriptional profile in response to treatment with UM-C162

**Figure 5:**
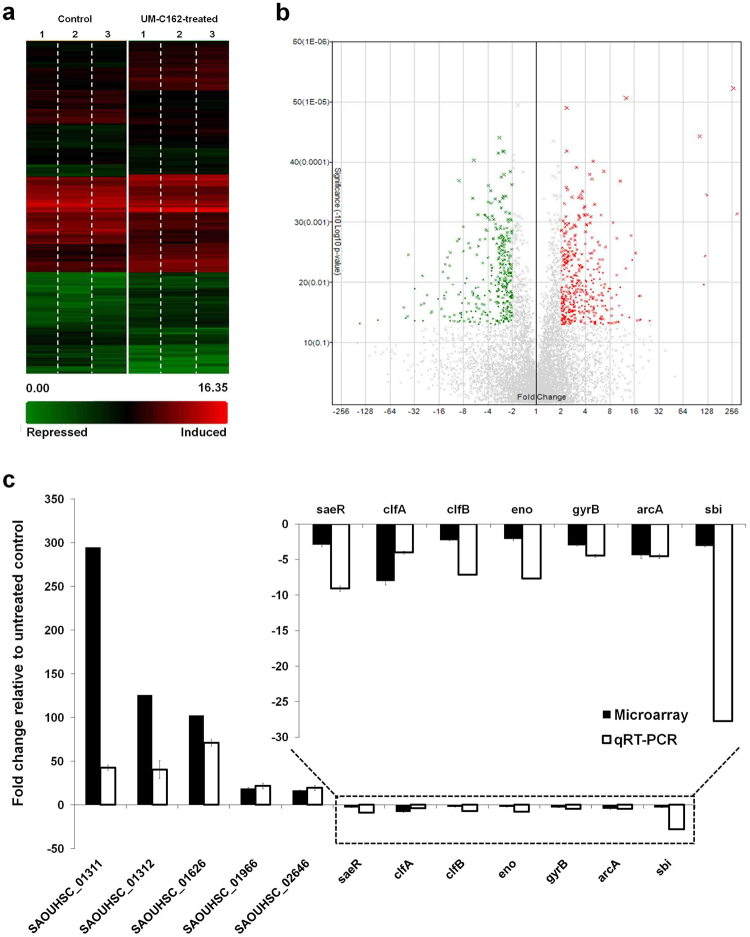
Transcriptome dynamics of UM-C162-treated and untreated *S*. *aureus*. (**a**) Hierarchically clustered expression profile (rows) for 456 *S*. *aureus* genes differentially expressed upon exposure to UM-C162 as compared to the untreated control. Data from three independent experiments (columns) are shown. (**b**) Volcano plot showing the gene level analysis to select differentially expressed genes after treatment with UM-C162. The data for all genes are plotted as fold change versus −log_10_ of the adjusted p-value. Green, grey and red correspond to genes with < −2-fold, −2 to 2 fold and > 2-fold differential expression, respectively. (**c**) Verification of microarray data by qRT-PCR. Transcript levels of twelve differentially expressed *S*. *aureus* genes from qRT-PCR and microarray derived data were compared. Results displayed are the average relative fold-change for transcripts from UM-C162-treated bacteria compared to untreated control. The relative quantification of *S*. *aureus* RNA was determined by the change in expression of genes of interest normalized to *S*. *aureus* 16 s rRNA housekeeping gene.

The expression of *S*. *aureus* virulence traits and components required for biofilm formation is tightly regulated by a complicated coordination of two-component systems and various transcriptional regulators^4^. Therefore, we postulated that a compound that can alter the formation of biofilm might also be able to reduce bacterial virulence. To probe the transcriptional changes of *S*. *aureus* in response to UM-C162, total RNA was isolated from the bacteria after overnight incubation in the presence of 6.25 µM UM-C162. Three independent microarray experiments were conducted to compare the gene expression profile of UM-C162-treated and untreated *S*. *aureus*. GeneChip analysis revealed a substantial number of genes (456) that were significantly modulated by UM-C162 (fold change ≤ −2 and ≥ 2, *p* < 0.05). Of these, 235 genes were up-regulated whilst 221 genes were down-regulated in UM-C162-treated *S*. *aureus* (Fig. 5a,b). A complete list of all genes differentially expressed by UM-C162 is available in Table S2. The microarray data is deposited in the NCBI Gene Expression Omnibus (GEO) (Accession number: GSE84485).

We validated the microarray derived expression data of a subset of genes by quantitative real-time PCR (qRT-PCR). The relative expression levels of five up-regulated genes and seven down-regulated genes were compared using RNA extracted from three independent bacterial cultures. A strong positive correlation was observed for the qRT-PCR and microarray derived data for all twelve genes tested, albeit with different magnitudes (Fig. 5c).

**Figure 6:**
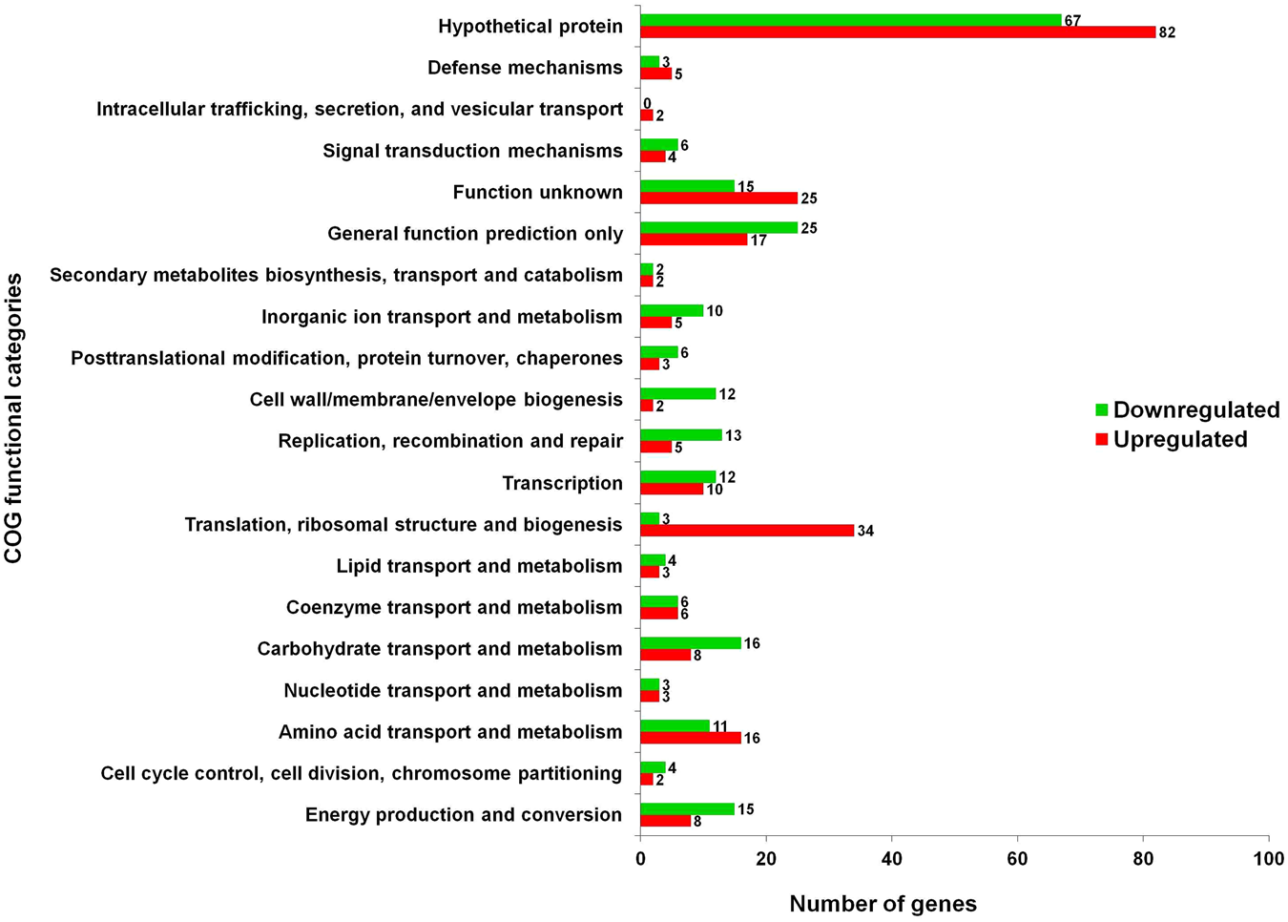
Functional classification of *S*. *aureus* genes significantly modulated by UM-C162. Genes were divided into their respective functional groups based on clusters of orthologous groups (COGs) designations. The red bars correspond to induced genes while green bars show the repressed genes when *S*. *aureus* was cultured in the presence of 6.25 µM UM-C162.The number at the end of each bar represents the total number of genes in each functional class.

The differentially expressed genes were classified into their respective functional groups using clusters of orthologous groups (COGs) designations. The distribution of UM-C162-modulated genes and their biological roles according to COGs classification is shown in Fig. 6. Of note, a majority of the genes were grouped as hypothetical proteins (32%), general function prediction only (9%) and genes with function unknown (8.7%). By comparing the number of up-regulated and down-regulated genes in each functional group, we noted that the group of translation-, ribosomal structure- and biogenesis-encoding genes contained a higher number of up-regulated genes (34) and only 3 down-regulated genes whilst genes involved in cell wall/membrane/envelope biogenesis and replication, recombination and repair were mainly down-regulated (12 and 13 genes, respectively) and only 2 or 5 up-regulated genes, respectively (Fig. 6). Other UM-C162-regulated genes were generally classified as genes involved in amino acid transport and metabolism, carbohydrate transport and metabolism, energy production and conversion, and transcription.

Further gene function enrichment analysis on the induced genes based on Gene Ontology (GO) Term identified significant enrichment (*p* < 0.0001) of genes involved in translation. In addition, KEGG pathway analysis showed enrichment of prokaryotic-type ATP-binding cassette (ABC) transporter/permease proteins (*p* < 0.001). On the other hand, the genes repressed by UM-C162 were mainly involved in cell wall/external encapsulating structure (*p* < 0.0001), virulence/pathogenesis (*p* < 0.0001) and response to stress/DNA repair (*p* < 0.01) (Table 1).

**Table 1:** Functional enrichment of *S*. *aureus* genes induced and suppressed by UM-C162.

Function	No. of genes	Genes	P value
**Up-regulated**			
Translation	30	SAOUHSC_02504, SAOUHSC_01666, *rpsC*, *rpsD*, *rpsE*, *rpsH*, *rpsJ*, *rpsQ*, *rpsS*, *rpsT*, *rplB*, *rplC*, *rplD*, *rplE*, *rplF*, *rplJ*, *rplN*, *rplP*, *rplQ*, *rplR*, *rplT*, *rplV*, *rplW*, *rplX*, *rpmA*, *rpmC*, *rpmD*, *def*, *pheT*, *thrS*,	*p* < 0.0001
ABC transporter/permease protein	8	SAOUHSC_00074, SAOUHSC_00136, SAOUHSC_00169, SAOUHSC_00693, SAOUHSC_01046, SAOUHSC_01311, SAOUHSC_01312, SAOUHSC_02821	*p* < 0.001
**Down-regulated**			
Cell wall/external encapsulating structure	23	SAOUHSC_00114, SAOUHSC_00356, SAOUHSC_00383, SAOUHSC_00386, SAOUHSC_00437, SAOUHSC_01127, SAOUHSC_02549, SAOUHSC_02708, SAOUHSC_02971, *capF*, *capL*, *capA*, *clfA*, *clfB*, *eno*, *hlgB*, *hlgC*, *sspA*, *sbi*, *hlb*, *sasA*, *sdrC*, *atl*	*p* < 0.0001
Pathogenesis/virulence	19	SAOUHSC_00383, SAOUHSC_00386, SAOUHSC_01127, SAOUHSC_02708, *clfA*, *clfB*, *eno*, *hlgB*, *hlgC*, *hlb*, *sspA*, *sbi*, *sasA*, *sdrC*, *saeR*, *yent1*, *arlS*, *clpL*, *clpC*	*p* < 0.0001
Response to stress/DNA repair	9	SAOUHSC_00503, SAOUHSC_01363, SAOUHSC_01667, *clpC*, *clpL*, *recO*, *recF*, *recA*, *recN*	*p* < 0.01

Genes encoding the ABC transporters SAOUHSC_01311 and SAOUHSC_01312 were among the most highly induced genes, with an induction magnitude of 294.62 and 125.66 fold, respectively (Table S2). ABC transporters are transmembrane proteins with diverse roles in cellular function, including the translocation of various substances (amino acids, metabolic products, lipids and drug molecules) across membranes^23^. Hence, the observed induction of ABC transporter genes may be associated with the active transport of UM-C162 molecules in and out of the bacterial cells. Exposure to UM-C162 also resulted in an up-regulation of ribosomal protein subunits encoded by *rps*, *rpl and rpm* (2 to 4-fold induction) (Table S2). Genes encoding ABC transporters and ribosomal proteins were also up-regulated in *S*. *aureus* exposed to ortho-phenylphenol, a phenolic compound with known antimicrobial properties^24^.

### UM-C162 represses the expression of biofilm-associated genes in *S*. *aureus*

To decipher the molecular mechanism underlying the ability of UM-C162 to inhibit *S*. *aureus* biofilm, we compared our list of differentially expressed genes to reported *S*. *aureus* biofilm-related genes^25–27^. Generally, many biofilm-related genes were indeed suppressed following *S*. *aureus* exposure to UM-C162 (Table 2). Specifically, genes encoding proteins that mediate cell adhesion and bacterial attachment (SAOUHSC_02690, *clfA*, *clfB*, *sdrC*, *sdrH*, *eno* and *arlS*) were among the major group of genes suppressed by UM-C162. The clumping factors (Clf) and serine-aspartate repeat-containing proteins (Sdr) are typical *S*. *aureus* microbial surface component-recognizing adhesive matrix molecules (MSCRAMMs) that promote adherence of bacteria to a substratum and induce staphylococcal biofilm formation^28^. Clumping factors enable the binding of bacterial cells to fibrinogen and have been identified as an important virulence determinant in various infection models^29,30^. Enolase (*eno*) mediates *S*. *aureus* pathogenesis by adhering to laminin-containing extracellular matrix^31^ and has also been shown to enhance bacterial invasiveness and metastasis to secondary sites of infection in other pathogens^32^. ArlS is a member of the two-component system ArlS-ArlR involved in the regulation of *S*. *aureus* adhesion, autolysis, multidrug resistance and production of virulence factors^33^. An *arlS* mutant exhibited increased biofilm production when cultured on a polystyrene surface; however, intercellular adhesion and cell aggregation properties of this mutant were impaired^34^. The abrogation of *arlS* promoted peptidoglycan hydrolase activity leading to cell autolysis. Thus, it was proposed that the *arl* system might be required for cell growth and division as well as bacterial adherence to polymer surfaces^34^. In general, the development of biofilm involves three key steps which are initial attachment, proliferation and maturation of biofilm, followed by detachment or dispersal of cells and dissemination of the bacteria^35^. Collectively, our findings propose that UM-C162 most likely blocks the primary attachment/adhesion during biofilm formation, preventing further development of the biofilm.

**Table 2:** Suppression of known *S*. *aureus* genes associated with biofilm formation.

Role in biofilm formation	Genes	Fold change (UM-C162-treated vs control)
Cell adhesion and bacterial attachment	SAOUHSC_02690	−3.39
	*clfA*	−8.03
	*clfB*	−2.25
	*sdrC*	−2.55
	*sdrH*	−2.56
	*eno*	−2.07
	*arlS*	−2.32
Capsular polysaccharide biosynthesis	SAOUHSC_00114	−2.91
	*capF*	−5.36
	*capL*	−3.83
	*capA*	−2.91
Intracellular multiplication	*clpB*	−9.54
	*clpC*	−5.23
	*ctsR*	−8.61
Surface protein of Gram positive cocci	SAOUHSC_00437	−3.69
	SAOUHSC_02549	−2.75
Teichoic acid biosynthesis protein	SAOUHSC_00974	−25.4
	SAOUHSC_00643	−2.6
Peptidoglycan biosynthesis	SAOUHSC_01987	−2.12

In addition to genes encoding cell adhesion and attachment, treatment of *S*. *aureus* with UM-C162 also led to a down-regulation of genes encoding for capsular polysaccharide (SAOUHSC_00114, *capF*, *capL* and *capA*), teichoic acid (SAOUHSC_00974 and SAOUHSC_00643) and peptidoglycan (SAOUHSC_01987) biosynthesis as well as Gram positive cocci surface proteins (Table 2). Among them, SAOUHSC_00974 encoding a poly (glycerol-phosphate) alpha-glucosyltransferase for teichoic acid biosynthesis was suppressed up to 25.4-fold. Polysaccharide capsule, cell wall teichoic acid, peptidoglycan and surface proteins are all essential constituents of the biofilm produced by Gram-positive bacteria such as *S*. *aureus* and *S*. *epidermidis*^35^. A reduction in the synthesis of these components is indicative of low biofilm extracellular polymeric substance production. From the analysis of the microarray data, we also noted reduced expression of ATP-dependent Clp proteases, *clpB* (−9.54-fold), *clpC* (−5.23-fold) (Table 2) and *clpL* (−2.64-fold) (Table S2). Inactivation of *clpC* in an *S*. *aureus* mutant decreased biofilm formation, proposing that ClpC is required for biofilm formation^36^. ClpC was also reported to affect *S*. *aureus* capsule production^37^. Furthermore, ClpB and ClpC, and to a minor extent ClpL, are required for bacterial long-term survival and intracellular multiplication in a cell-culture based assay^36^. However, we also noted that the expression of *ctsR*, a negative regulator of *clp* expression^38^, was repressed (−8.61-fold) in UM-C162-treated samples. Hence, *clp* expression may be regulated by more than one regulator or pathway.

### UM-C162 diminishes the production of key virulence factors in *S*. *aureus*

**Figure 7:**
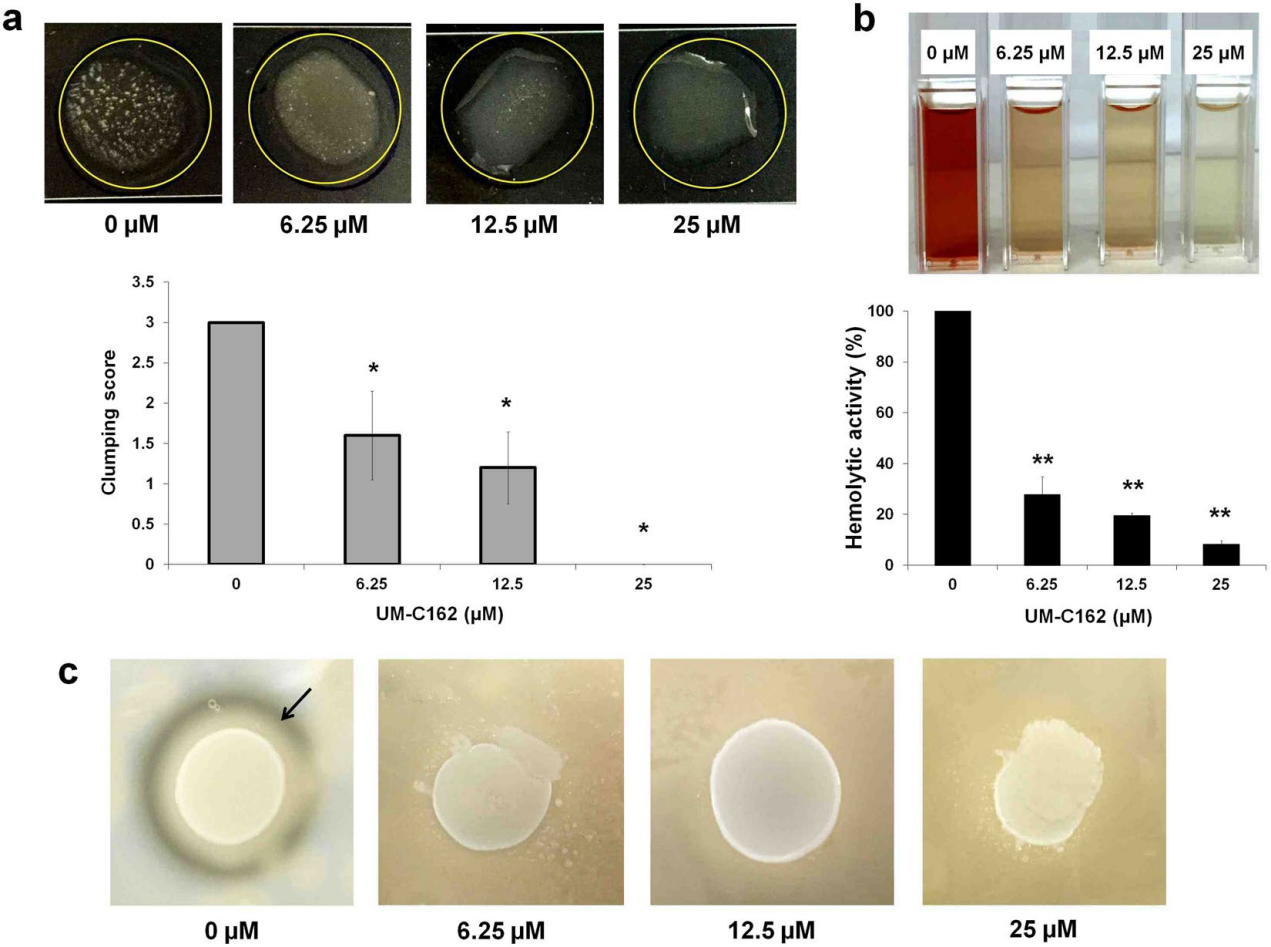
Effect of UM-C162 on *S*. *aureus* virulence factors. (**a**) Results of *S*. *aureus* slide clumping factor assay. Shown are the pictures of *S*. *aureus* cell clumping in rabbit plasma in the presence and absence of UM-C162. Cultures supplemented with UM-C162 at 6.25 µM and 12.5 µM demonstrate weak agglutination with turbid background while cells treated with 25 µM show no cell agglutination. Bars represent the mean ± SD of clumping scores (expressed in arbitrary units) corresponding to the formation of bacteria cell clumps (n = 5 for each treatment) according to the criteria presented in Fig. S5. (**b**) Anti-hemolytic property of UM-C162 against *S*. *aureus*. Hemolysis of red blood cells by *S*. *aureus* in the presence of UM-C162 at 6.25 µM, 12.5 µM and 25 µM was measured following 4 hours incubation at 37 °C. Treatment with UM-C162 at various concentrations dramatically reduces the ability of *S*. *aureus* to lyse red blood cells. Pictures of spectrophotometer cuvettes indicating hemolytic activity of *S*. *aureus* are shown. (**c**) Effect of UM-C162 on the production of *S*. *aureus* proteases. Shown are the representative micrographs of halo formation (indicated by black arrow) in untreated *S*. *aureus* culture (0 µM) and absence of halo formation in UM-C162-treated bacteria on 3% skim milk agar after 24 hours incubation. (*) marks significant difference between untreated (0 µM) and UM-C162-treated bacteria at *p* < 0.05, (**) *p* < 0.01.

Biofilm has been recognized as a major bacterial virulence factor. The components that contribute to the formation of biofilm, for example, *clf*, *eno*, *clp* and *cap* are also classified as important virulence factors for *S*. *aureus*. ClfA mediates attachment to plasma clots and platelets whilst ClfB enhances clumping of cells in the presence of fibrinogen^39^. We noted that both *clfA* and *clfB* transcript levels in UM-C162 treated *S*. *aureus* were reduced relative to the untreated bacteria. To validate the ability of UM-C162 to inhibit *S*. *aureus* clumping factors, we assessed bacterial cell clumping activity using the slide coagulation test with rabbit plasma. Scores from 0 to 3 + were assigned based on the ability of *S*. *aureus* cells to form macroscopic clumps on the slides (Fig. S5). In the absence of UM-C162, *S*. *aureus* consistently agglutinated in rabbit plasma, whereas in bacteria-treated with UM-C162, less clumping activity was observed (*p* < 0.05) (Fig. 7a). The aggregates or clumps formed on the slides decreased with increasing concentration of UM-C162 and treatment with 25 µM UM-C162 completely abolished the clumping activity of *S*. *aureus* (Fig. 7a).

*S*. *aureus* produces a series of hemolysins including γ-hemolysin which lyse rabbit erythrocytes and various leukocytes such as neutrophils, monocytes, granulocytes and macrophages^40^. γ-hemolysin corresponds to two functional bi-component pore-forming toxins, HlgAB and HlgCB, which share the common HlgB protein. From the transcription profile, there is a significant decrease in the expression of genes encoding the bi-component γ-hemolysins (*hlgA* (−3.63 fold), *hlgB* (−4.52 fold) and *hlgC* (−6.61 fold)) (Table 3) in UM-C162-treated bacteria when compared to untreated bacteria. The *hlb* gene encoding ß-hemolysin or phospholipase C and SAOUHSC_02708, a putative leukocidin, were also down-regulated in UM-C162-treated *S*. *aureus* whilst the α-toxin (*hla*) was not significantly modulated by UM-C162. To validate the suppression of *S*. *aureus* hemolysin production by UM-C162, rabbit erythrocytes were exposed to UM-C162 treated bacteria. We noted that UM-C162 treated *S*. *aureus* exhibited weak hemolytic activity (Fig. 7b). Up to 70% inhibition in hemolytic activity was noted upon treatment with UM-C162 from as low as 6.25 µM (*p* < 0.01) (Fig. 7b), confirming a disruption in hemolysin production by *S*. *aureus*. Treatment with UM-C162 at 12.5 µM and 25 µM led to a greater degree of hemolysis inhibition of 80% and 92% inhibition respectively.

**Table 3:** Down-regulation of *S*. *aureus* genes encoding major virulence factors.

Virulence factors	Genes	Fold change (UM-C162-treated vs control)
Hemolysins/leukocidin	*hlgA*	−3.63
	*hlgB*	−4.52
	*hlgC*	−6.61
	SAOUHSC_02708	−3.63
Phospholipase C	*hlb*	−2.31
Serine protease (V8 protease)	*sspA*	−2.07
Staphylococcal superantigen-like (SSL) proteins	SAOUHSC_00383	−2.05
	SAOUHSC_00386	−2.78
	SAOUHSC_01127	−2.13
Immunoglobulin-binding protein	*sbi*	−3.09
*S*. *aureus* surface protein A	*sasA*	−2.84
*S*. *aureus* exoprotein expression protein	*saeR*	−2.9

In addition to ATP-dependent Clp proteases (Table 2), expression of *sspA* encoding a V8 serine protease, was also reduced in UM-C162-treated samples as compared to untreated bacteria (Table 3). Clp proteases direct cellular functions through proteolysis of proteins such as casein and albumin, in the presence of ATP. In *S*. *aureus*, virulence of *clp* mutants is impaired in animal infection models^36^, suggesting that Clp proteases are crucial for normal cellular physiology as well as the control of the pathogen virulence machinery. V8 protease is thought to facilitate host tissue invasion as well as nutrient acquisition. The importance of *S*. *aureus* V8 protease for bacterial survival and virulence *in vivo* has been revealed in a number of animal infection models^41^. To assess the effect of UM-C162 on the production of *S*. *aureus* proteases, we performed the skim milk agar-based protease assay for both treated and untreated bacterial cultures. In the absence of UM-C162, *S*. *aureus* proteases hydrolyzed the milk casein resulting in the formation of a clear zone around the bacterial colony (Fig. 7c). When the bacteria were grown in the presence of various concentrations of UM-C162, no halo formation was evident (Fig. 7c), proposing that UM-C162 prevented the secretion of proteases by *S*. *aureus*.

In addition to hemolysins, proteases, clumping factors and the virulence factors involved in biofilm formation, treatment with UM-C162 also resulted in reduced expression of other virulence-related genes, including staphylococcal superantigen-like (SSL) proteins (SAOUHSC_00383, SAOUHSC_00386 and SAOUHSC_01127), immunoglobulin-binding protein (*sbi*), *S*. *aureus* surface protein A (*sasA*) and *S*. *aureus* exoprotein expression protein (*saeR*) (Table 3). Taken together, these biochemical tests validate the proposal that UM-C162 is a promising anti-biofilm and anti-virulence agent towards *S*. *aureus*.

## Discussion

*S*. *aureus* is a key etiological agent responsible for infections that impose substantial financial burden on hospitals globally as well as high mortality and morbidity rates. The alarming increase in antibiotic-resistant *S*. *aureus* advocates the need to search for novel anti-infective drugs. In this study, we screened a collection of 35 benzimidazole derivatives for potential anti-infective activity towards *S*. *aureus*^16^. From this screen, we identified 12 positive hits of which three are able to alleviate *S*. *aureus* biofilm formation. Previously, a similar screen successfully identified a number extracts and compounds with anti-bacterial effects^16^. In addition, a plant extract that did not affect bacterial growth was able to stimulate the host antimicrobial response to clear the infection^42^. Hence, it is possible that some of the positive hits may permit increased survival of infected hosts by inhibiting bacterial growth or activating the host immune response to eradicate the bacteria. Benzimidazoles are widely used as anti-helminthics to treat nematode and trematode infections in domestic animals^43^. In our study, we also noted a small number of benzamidazole derivatives that killed *C*. *elegans* even in the absence of infection supporting the advantage of this *C*. *elegans* screen in facilitating the elimination of compounds that are toxic to the host.

Rapid developments and advances in structure-based rational design of novel molecular entities that function or target certain biomolecular pathways have led to the discovery of potential therapies for infectious diseases. The collection of benzimidazole derivatives used in this study have previously been tested for their inhibitory effect on poly (ADP-ribose) polymerase-1 (PARP-1) and dihydroorotate dehydrogenase (DHODH) and a number of these compounds showed promising anti-PARP-1 and DHODH effects, suggesting their prospective role in anti-cancer therapy^44^. Of note, two positive hits in our anti-infective screen, UM-C49 and UM-C201, were also able to inhibit both PARP-1 and DHODH enzymes. UM-C162 demonstrated modest DHODH inhibition and a compound with a similar structure (UM-C187) showed dual potency on both DHODH and PARP-1^44^. Recently, several other compounds that exhibit both anti-cancer and anti-biofilm effects *in vitro* have been discovered^45,46^. Nevertheless, the link between anti-cancer and anti-biofilm activities is yet to be clarified.

An anti-infective agent that reduces bacterial virulence without disrupting bacterial viability is thought to avoid the selective pressure that promotes antibiotic resistance^5^. Benzimidazole derivatives with potent antimicrobial activity against both bacteria and fungus have previously been reported^15^. Conversely, in our study, both the antimicrobial test (Fig. S4) and growth curve analysis (Fig. 4a) demonstrated that UM-C162 was not antibacterial towards *S*. *aureus*, advocating the potential of UM-C162 as an anti-virulence candidate that is not likely to stimulate bacterial resistance. Furthermore, an anti-virulence agent that does not impede bacterial growth is not likely to interfere with the preservation of host endogenous flora.

Previously, a benzimidazole analog, 5-methoxy-2-[(4-methylbenzyl)sulfanyl]-1*H*-benzimidazole (also known as ABC-1), was identified as a wide-spectrum biofilm inhibitor towards both Gram-positive and Gram-negative bacterial biofilm (including *S*. *aureus*, MRSA, *Vibrio cholerae*, *Pseudomonas aeruginosa*) without having any impact on bacterial growth^18^. At 25 µM, ABC-1 inhibited *S*. *aureus* biofilm by < 50% whilst in this study, UM-C162 was able to inhibit *S*. *aureus* biofilm by > 50% even at 6.25 µM. In addition, the 2-aminobenzimidazole class of compounds also exhibits biofilm inhibitory effects^45^, proposing that compounds containing a benzimidazole functional group are a promising scaffold for the discovery of novel anti-biofilm candidates.

The diversity and functional redundancy of *S*. *aureus* virulence factors is important to the overall fitness and disease causation of *S*. *aureus* in the host. As a result, attenuating a single virulence factor does not usually lead to the diminution of *S*. *aureus* virulence. Our genome-wide transcriptome profile and SEM analysis indicate that UM-C162 most likely blocks *S*. *aureus* adherence or cell aggregation, which is the first step in the process of biofilm production. A cohort of virulence factor-encoding genes was suppressed in UM-C162-treated bacteria but not in the untreated control. Together with the outcomes from the biochemical assays, we provide evidence that compound UM-C162 inhibits the production of several *S*. *aureus* virulence factors, namely biofilm, clumping factors, hemolysins and proteases. These surface proteins, toxins and enzymes are vital for the pathogen to attach to host cells, establish an infection, impair the host immune reaction and result in host tissue deterioration. A previous *S*. *aureus - C*. *elegans* host-pathogen interaction study revealed that the virulence determinants, including hemolysins, V8 serine proteases and biofilm, are needed for full pathogenicity of *S*. *aureus* in the nematode model of infection^17,47^. *S*. *aureus* mutants for these crucial virulence factors were attenuated in *C*. *elegans* killing^47^. By blocking these virulence traits, the bacteria are rendered less virulent and less able to disseminate within the host. This allows the host immune response to initiate elimination of the less pathogenic bacteria from the host, ensuring the survival of the infected host.

A complex process of multiple systems is required to regulate the expression of virulence-associated genes. Among the well-studied regulators are the two component systems Agr operon, SaeRS, ArlSR and SarA global transcriptional regulators^4^. We noted a down-regulation of the transcript levels for *saeR* and *arlS* while the expression of *agr* and *sarA* remained unchanged upon treatment with UM-C162, suggesting that UM-C162 might target the regulator/s upstream of saeRS and arlSR. SaeRS and ArlSR regulate the expression of genes encoding *S*. *aureus* virulence factors such as hemolysins, coagulases, lipase and *S*. *aureus* surface protein A^48,49^. Through various biochemical assays, we consistently observed suppression of hemolysins, coagulases and proteases by UM-C162.

*S*. *aureus* biofilms are often associated with chronic infections and contaminated implanted medical devices and the presence of biofilm renders the bacteria highly tolerant to antibiotics and able to resist phagocytosis. Additionally, *S*. *aureus* expresses an arsenal of virulence determinants that contribute to its versatility in surviving the host defence response. In our search for novel anti-infectives that target bacterial virulence, we identified a benzimidazole-based molecule, UM-C162, as a potential anti-virulence agent that interferes with the production of bacterial virulence determinants (e.g. toxins, enzymes and biofilm). In light of its ability to inhibit a myriad of key *S*. *aureus* virulence factors, UM-C162 is a promising anti-virulence agent to be further developed as an anti-infective agent. No detectable toxic effect on adult worms was observed signifying that UM-C162 is non-toxic to the eukaryotic host. Our results strongly propose UM-C162 as a potential anti-virulence candidate with minimal toxicity, to be developed as an alternative therapeutic for *S*. *aureus* infections.

## Methods

### Bacteria and nematode growth conditions

*S*. *aureus* strains used were NCTC8325-4, ATCC25923 and a MRSA clinical strain^22^ (Adelaide Pathology Partners (Mile End, Australia)). *S*. *aureus* NCTC8325-4 was used in all experiments unless otherwise specified. *S*. *aureus* was routinely grown in Trypticase Soy (TS) media (Oxoid/Pronadisa) and incubated at 37 °C under aerobic conditions. A uracil-requiring strain of *Escherichia coli* OP50 was propagated on Luria-Bertani (LB) media (Pronadisa) with streptomycin (100 µg/mL) at 37 °C. The wild type *C*. *elegans* N2 strain was cultivated on nematode growth medium (NGM) with *E*. *coli* OP50 as their food. The nematodes were rendered sterile by RNAi knockdown of the *pos-1* gene that caused the gravid worms to lay unhatched eggs^50^. This eliminates any confusion in the scoring as a result of nematode reproduction. The *pos-1* RNAi treated nematodes were allowed to develop into young adult worms to be used in the screen.

### Synthesis and preparation of benzimidazole derivatives

**Figure 8:**
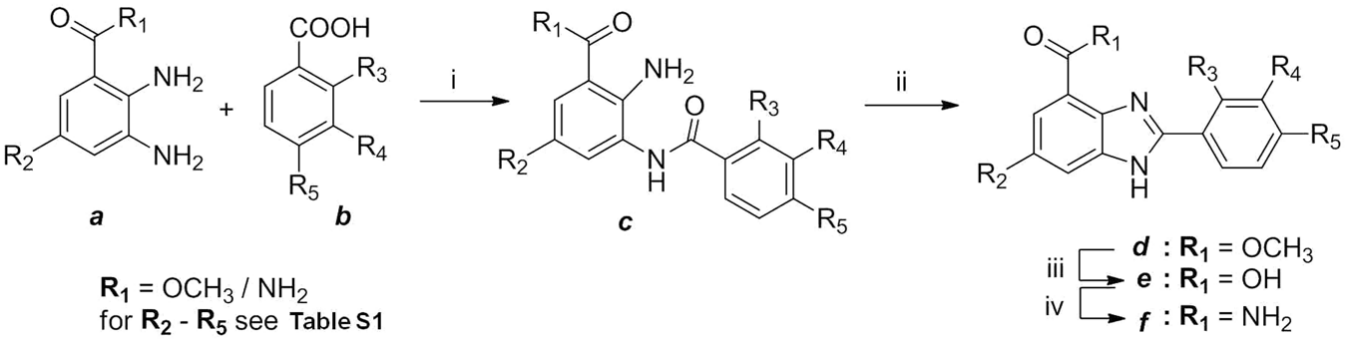
General synthesis of benzimidazole derivatives. Reaction conditions: For R_1_ = OCH_3_ (i) HATU, DIPEA, DMF (ii) AcOH, 110 °C (iii) LiOH, MeOH/THF (iv) HOBt, EDCl, NH_4_Cl, DIPEA, DMF; For R_1_ = NH_2_ (i) CDI, pyridine, DMF (ii) AcOH, 110 °C.

Thirty five compounds with the benzimidazole backbone were synthesized using the previously described procedures^44^ as outlined in the scheme in Fig. 8. Briefly, various functional groups were placed at the R_3_, R_4_ and R_5_ positions of the corresponding carboxylic acid intermediates by de novo synthesis. Two different routes were employed to prepare the compounds. The first route (for R_1_ = OCH_3_) involves coupling of diamine ***a*** with carboxylic acid ***b*** by using 1-[bis(dimethylamino) methylene]-1*H*-1,2,3-triazolo[4,5-b]pyridinium-3-oxidhexafluoro-phosphate (HATU). Subsequent thermal cyclization of the resulting amide ***c*** in acetic acid gave benzimidazole ester ***d***. Saponification of the ester ***d*** with lithium hydroxide resulted in the benzimidazole carboxylic acids ***e*** which were then converted to the corresponding benzimidazole carboxamide ***f*** with 1-hydroxybentzotriazole (HOBt). The second route (for R_1_ = NH_2_) involves direct coupling of diamine ***a*** with carboxylic acid ***b*** in the presence of 1,1′-carbonyldiimidazole (CDI). Subsequent thermal cyclization of the resulting amide ***c*** in one-pot afforded benzimidazole carboxamide ***f*** in two-steps. DMSO was used as the solvent and all reconstituted compounds were filter sterilized using a 0.2 µm membrane and stored at −20 °C before use.

### *C*. *elegans* – *S*. *aureus* anti-infective screen

Screening for potential anti-infective agents was performed as previously described^16^. Briefly, medium containing worm M9 buffer, *S*. *aureus* culture and 10 µg/mL cholesterol was prepared and added into individual wells in a 48-well plate. Each benzimidazole derivative was individually added to a final concentration of 100 µM. The controls were prepared by replacing the compound with DMSO (untreated control) or by replacing *S*. *aureus* with *E*. *coli* OP50 (uninfected control). Ten age-matched *pos-1* treated nematodes were manually transferred into the liquid medium and the plate was incubated at 25 °C without agitation. The number of live and dead worms was scored every 24 hours. Positive hits were selected based on ≥70% survival of *S*. *aureus*-infected compound-treated worms in at least two replicates at a point when ~20% of the untreated control worms survived.

### Antimicrobial tests

To determine the antimicrobial activity of benzimidazole compounds, the broth microdilution test and bacterial growth analysis were performed. *S*. *aureus* was cultured in 3 mL of TS broth and incubated overnight (16–18 hours) at 37 °C in a shaking incubator. The initial bacterial inoculum size was standardized by diluting the *S*. *aureus* overnight culture to OD_600_ = 0.3. The bacterial suspension was further diluted 1:100 to obtain 10^6^ cfu/mL. To determine the minimum inhibitory concentration (MIC), a series of different concentrations of compound was prepared by two-fold serial dilution in a 96-well plate, followed by inoculation with 10^6^ cfu/mL *S*. *aureus* and overnight incubation at 37 °C. Gentamicin (0.03 mg/mL to 50 mg/mL) and DMSO (0.0005% to 1%) served as the positive and negative controls, respectively. To evaluate the effect of compound UM-C162 on *S*. *aureus* growth, a standardized suspension of 10^8^ cfu/mL *S*. *aureus* was diluted (1:100) in TS broth supplemented with the compound at different concentrations, followed by incubation at 37 °C with shaking. At selected time intervals, the turbidity of the culture was measured at 600 nm.

### Crystal-violet biofilm assay

The biofilm assay was conducted in a 96-well polystyrene flat-bottomed microplate (Greiner Bio-One). Firstly, the standardized *S*. *aureus* suspension at 10^6^ cfu/mL was prepared and dispensed into each well in the 96-well plate. To screen for anti-biofilm activity, individual benzimidazole compounds were added to the bacterial suspension to a final concentration of 100 µM and tested in duplicate wells, representing two technical replicates. For the dose-dependent anti-biofilm assay, the selected compound was tested across of series of concentrations, ranging from 100 to 0.78 µM. As a control, the compound was replaced with DMSO. Uninoculated TS broth was also included as the blank control. The plate was incubated at 37 °C for 24 hours under static conditions. After incubation, the wells were washed three times with 1X phosphate buffered saline (PBS) to eliminate non-adherent bacteria and fixed with 99% (v/v) methanol for 15 minutes. The wells were allowed to dry in a laminar flow. The attached biofilm cells were stained using filtered 0.5% crystal violet (Sigma-Aldrich) for 5 minutes at room temperature. The excess stain was removed by rinsing with water and crystal violet bound cells were solubilised with 33% acetic acid. The released stain was measured at 570 nm using a microplate reader (Sunrise, Tecan). Analysis was performed on three independent occasions and two technical replicates for each compound.

### Scanning electron microscopy (SEM) analysis of biofilm formation

An overnight culture of *S*. *aureus* was adjusted to OD_600_ = 0.3 and further diluted 1:100 in TS broth to obtain 10^6^ cfu/mL *S*. *aureus*. UM-C162 was tested at 6.25 µM, 12.5 µM and 25 µM. For each concentration, 4 mL of bacterial suspension was supplemented with the desired amount of compound and added into individual wells in a 6-well plate. A glass slide (about 10 mm × 10 mm) was positioned in the middle of each well. The plate was incubated at 37 °C for 24 hours to allow the formation of biofilm on the slides. The bacteria cells were processed for SEM as described previously^51^. Briefly, the samples were fixed overnight at 4 °C in 4% (v/v) glutaraldehyde, rinsed three times with 1X PBS and dehydrated through a graded ethanol series (35%, 50% and 70%), followed by critical-point drying in a drying apparatus with liquid CO_2_. The samples were gold-coated using a gold sputtering unit and observed using a LEO 1450VP variable pressure scanning electron microscope (Electron Microscopy Unit, Universiti Kebangsaan Malaysia).

### Artificial dermis wound biofilm model

An artificial dermis was prepared as previously described^22^. The dermis consisting of hyaluronic acid and collagen spongy sheets was sterilized by UV light treatment followed by heating at 110 °C for an hour before use. A mixture of lyophilized bovine plasma, Bolton broth, horse blood and heparin was prepared and added to the dermis in a 24-well plate. Overnight cultures of *S*. *aureus* ATCC 25923 and a clinical strain of MRSA was adjusted to 1 × 10^6^ cfu/mL and 10 µL of adjusted bacterial inoculum was inoculated onto the surface of each dermis. To assess for a potential biofilm inhibitory effect, UM-C162 was added to each artificial dermis immediately after the dermis was inoculated with bacteria. On the other hand, to assess for the eradication of biofilm, following bacterial inoculation, biofilm was allowed to form on the dermis at 37 °C for 24 hours. After the formation of biofilm on each dermis, sterile filter paper impregnated with UM-C162 was placed in contact with the biofilm, followed by incubation at 37 °C for 24 hours. The dermis was transferred to 10 mL saline and biofilm was extracted by vortexing and sonication. Recovered bacterial cells were serially diluted and plated on TSA supplemented with 7.5% NaCl. Plates were incubated overnight at 37 °C and colonies were counted. All experiments were repeated twice independently with at least two technical replicates.

### Nematode survival and lifespan assays

*C*. *elegans* survival and lifespan assays were conducted to examine the effect of benzimidazole compounds in a live-animal model as previously described^16,42^. The survival assay was performed to confirm the anti-infective effect of the selected benzimidazole compound, UM-C162, at different concentrations whilst the lifespan assay was performed to assess potential toxicity on uninfected worms. These assays were performed using a liquid-based medium in the same manner as the anti-infective screen except the number of worms used was increased from ten to twenty worms per well and six technical replicates were analysed. The lifespan assessment experiment was conducted by feeding the worms with heat-killed *E*. *coli* OP50 in the presence of UM-C162. Heat-killed (65 °C for 30 minutes) *E*. *coli* OP50 were used to avoid any possible effect of UM-C162 on the bacteria which may contribute to a reduction in *C*. *elegans* lifespan. The bacteria were added into M9 medium supplemented with different concentrations of compound before transferring age-matched *pos-1* RNAi treated nematodes into the medium. The plates were kept at 25 °C. Survival of treated and untreated worms was monitored over time until all worms died. The experiment was performed in duplicate.

### Total RNA extraction

Total bacterial RNA was extracted using MasterPure^TM^ Complete DNA and RNA Purification Kit (Epicentre) with minor modifications. In brief, *S*. *aureus* was cultured for 16–18 hours in the presence and absence of UM-C162 (6.25 µM) at 37 °C with agitation. The bacterial culture was centrifuged at 8000 × *g* for 1 minute to pellet the cells which were then resuspended in 150 µL of TE buffer (Epicentre MasterPure^TM^ Complete DNA and RNA Purification Kit). Lysozyme (15 µL) (Sigma-Aldrich) was added to a final concentration of 2.5 mg/mL and the mixture was incubated at 37 °C for 30 minutes. Ten µL of Proteinase K (Qiagen) was diluted in 150 µL of Tissue & Cell Lysis Solution and added to the samples, followed by incubation at 65 °C for 15 minutes in a water bath. After incubation, the samples were left to equilibrate to room temperature and placed on ice for 3 minutes. Removal of contaminating DNA was done according to Epicentre protocols. Total RNA was further purified with the Qiagen RNeasy Mini Kit and residual DNA was completely removed using the Qiagen RNase-Free DNase Set according to the manufacturer’s instructions. The presence of DNA contamination in the RNA samples was assessed by PCR targeting the *nuc* gene using primers and PCR protocol as previously described^52^. The quantity and quality of RNA was measured with the NanoDrop 2000C spectrophotometer and RIN number was analyszed using the Agilent 2100 Bioanalyser.

### Microarray gene expression profiling and data analysis

RNA samples were processed according to the Affymetrix SensationPlus FFPE Amplification and 3′IVT Labeling Kit protocol. Briefly, 50 ng of total RNA was reversed transcribed to produce cDNA/mRNA hybrid molecules, which were subsequently used as template to produce sense RNA via *in vitro* transcription (IVT). IVT-generated sense RNA was purified, subjected to single-stranded cDNA synthesis following which the cDNA was fragmented, end-labelled and hybridized to the Affymetrix GeneChip *S*. *aureus* Genome Array (Affymetrix, Cat. No. 900514) for 16 hours at 45 °C with rotation at 60 rpm. Following hybridization, arrays were washed and stained using the FS450_0005 fluidics protocol and scanned using an Affymetrix 3000 7 G scanner. The scanned images were inspected for hybridization efficiency. To perform data quality control (QC), feature intensity (CEL) files generated from GeneChip® Command Console® Software (AGCC) were imported into the Affymetrix Expression Console (EC) 1.4 software. MAS5 algorithm was used for normalizing probe-level intensity. QC metrics were generated and extracted from the Affymetrix EC report. Raw data were submitted to the Affymetrix EC software to generate probe set summarization (CHP) files from CEL files using the PLIER algorithm. Gene expression analysis was performed using three independent mRNA samples for each group. To perform statistical analysis and identify the differentially expressed genes, the resultant data (CHP files) were submitted to the Affymetrix® Transcriptome Analysis Console (TAC) software for Gene Level Differential Expression Analysis. Genes with a *p* value < 0.05 and threshold values of ≥ 2 and ≤ – 2-fold change between the UM-C162-treated samples and the untreated controls were defined as significantly differentially expressed. The complete microarray dataset has been deposited in NCBI’s GEO database and can be accessed through GEO accession number GSE84485 (http://www.ncbi.nlm.nih.gov/geo/query/acc.cgi?acc=GSE84485). Functional classifications were carried out based on clusters of orthologous groups (COGs) designations for *S*. *aureus* NCTC8325 (http://www.ncbi.nlm.nih.gov/COG/). Gene function enrichment analysis was performed against the Database for Annotation, Visualization and Integrated Discovery (DAVID) Bioinformatics Resources version 6.7 (https://david.ncifcrf.gov/) using Fisher Exact test with Benjamini-Hochberg multiple testing correction (*p* < 0.05).

### Quantitative RT-PCR (qRT-PCR) analysis

qRT-PCRs on total DNA-free RNA using the iTaq^TM^ Universal One-Step RT-qPCR kits with SYBR Green (BioRad Laboratories, USA) were performed according to the manufacturer’s recommendations on the Bio-Rad CFX96 Touch Real-Time PCR Detection System. The parameters for the RT-PCR cycle used were 50 °C for 30 minutes, 95 °C for 5 minutes, 95 °C for 30 seconds, 60 °C for 30 seconds, 76 °C for 30 seconds and 60 °C for 7 minutes. Amplification specificity was confirmed by melt curve analysis where the reaction temperature was increased from 55 °C to 95 °C over 10 seconds (0.5 °C increment for 80 cycles) after amplification. The forward and reverse primers used are listed in Table S3. The average cycle threshold (C_t_) values were normalized against the *S*. *aureus* 16 s rRNA^53^ that was found to not vary upon treatment. The mRNA expression levels of genes of interest was computed using the CFX Manager^TM^ software (Bio-Rad Laboratories, USA) on the average normalized C_t_ value.

### Analysis of virulence factors production

#### Plasma clumping factor test

*S*. *aureus* was cultured overnight at 37 °C in the presence and absence of UM-C162. Bacterial cells harvested from the overnight culture were prepared in 1X PBS. Rabbit blood plasma was separated from red blood cells by centrifugation at 900 × *g* for 5 minutes. Twenty µL of rabbit plasma was placed on the surface of a glass slide, followed by inoculation of 10 µL of *S*. *aureus* suspension. The mixture was mixed evenly for 10 seconds and macroscopic clumping/agglutination of bacterial cells was scored from 0 to 3+ (Fig. S5).

#### Haemolysin test

Lysis of red blood cells was assessed as previously described^9^ with minor modifications. Briefly, different concentrations of UM-C162 were added to a *S*. *aureus* culture with standardized inoculum size, followed by incubation at 37 °C with agitation for 16–18 hours. Rabbit blood was obtained from the Animal House Facility, Universiti Kebangsaan Malaysia. Red blood cells were separated from plasma by centrifugation at 900 × *g* for 5 minutes at 4 °C, washed 3 times with 1XPBS and diluted in PBS (330 µL of red blood cells in 10 mL of 1X PBS). To measure the haemolytic activity, 200 µL of *S*. *aureus* culture (OD_600_ = 0.3) grown in the presence of compound was added into 10 mL of diluted red blood cells. The negative control (*S*. *aureus* supplemented with DMSO only) was run in parallel. The mixture was incubated shaking for 4 hours. Supernatants were collected by centrifugation at 16,600 × *g* for 10 minutes and the OD readings were measured at 543 nm.

#### Protease test

The ability of UM-C162-treated and untreated *S*. *aureus* to produce protease was determined by spotting the bacterial culture on 3% skim milk agar (Oxoid, UK). *S*. *aureus* was grown overnight at 37 °C in the presence and absence of compound and adjusted to the standard inoculum size. Ten µL of standardized *S*. *aureus* inoculum was spotted onto skim milk agar, allowed to dry under sterile conditions and incubated at 37 °C for 24 hours. After 24 hours incubation, plates were observed for the formation of a clear zone/halo around the bacteria.

### Statistical analysis

At least three replicates were performed for the purpose of statistical analysis unless otherwise specified. For assays involving enumeration of dead and live worms, the worms were defined as dead when no response was detected upon picker touch and no pumping was observed at the pharynx. Dead worms with protruding vulvas were excluded from the analysis. Data from survival assays were analysed using the Kaplan-Meier nonparametric analysis in the StatView® 5.0.1 software (SAS Institute, Inc.). The pair wise comparison was analysed using the Log-rank (Mantel-Cox) significance test. For selection of significantly differentially expressed genes in the microarray analysis, a One-Way Between-Subject Analysis of Variance (ANOVA) (unpaired) was performed for treated and untreated groups. The data from other experiments were expressed as mean ± standard error of mean from at least two independent assays or mean ± standard deviation from one representative replicate. Statistical analyses for the biofilm assays and hemolysin test were performed using Student’s *t*-test. The ordinal data from the clumping factor assay were analyzed with the Mann-Whitney U-test.

## Electronic supplementary material

Supplementary Information
