# Supplementary material for: Suppression of Staphylococcus aureus biofilm formation and virulence by a benzimidazole derivative, UM-C162
Source: Sci Rep. 2018 Feb 9;8:2758. doi: 10.1038/s41598-018-21141-2 (PMC5807447; doi:10.1038/s41598-018-21141-2)
Supplement: Supplementary file 1 — Supplementary Information [file 41598_2018_21141_MOESM1_ESM.pdf]

Supplementary Information

Suppression of *Staphylococcus aureus* biofilm formation and virulence by a benzimidazole derivative, UM-C162

Cin Kong, Chin Fei Chee, Katharina Richter, Nicky Thomas, Noorsaadah Abd. Rahman & Sheila Nathan

Table S1. Details of all benzimidazole compounds used in anti-infective screen.

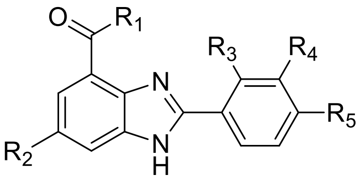

General Scheme

No.	ID	Molecular weight	R_1	R_2	R_3	R_4	R_5	Molecular structure
1	UM-C12	313.12	NH_2	H	H	H	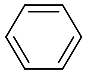	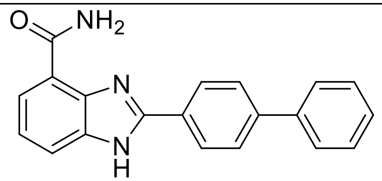
2	UM-C13	327.14	NH_2	CH_3	H	H	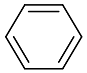	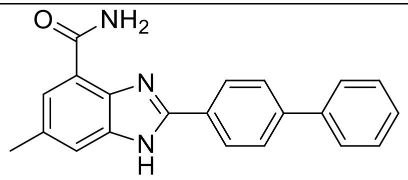
3	UM-C14	267.10	NH_2	H	H	H	OCH_3	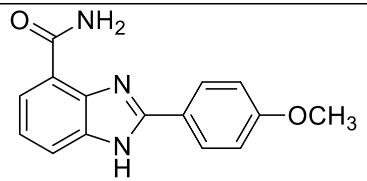

4	UM-C15	281.12	NH_2	CH_3	H	H	OCH_3	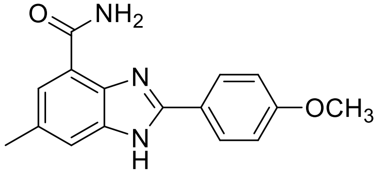
5	UM-C16	305.08	NH_2	H	H	H	CF_3	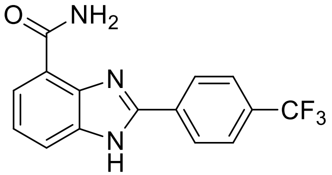
6	UM-C17	319.09	NH_2	CH_3	H	H	CF_3	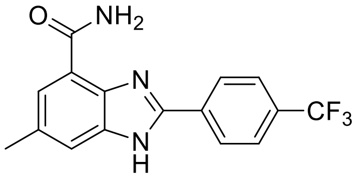
7	UM-C19	276.10	NH_2	CH_3	H	H	CN	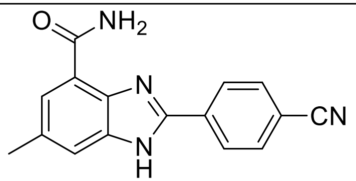
8	UM-C41	329.13	NH_2	H	H	H	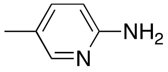	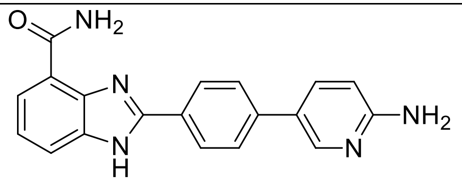
9	UM-C42	314.12	NH_2	H	H	H	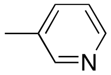	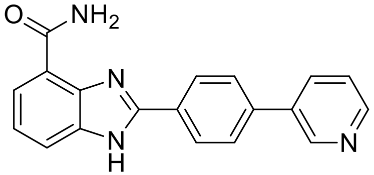

10	UM-C43	411.17	NH_2	H	H	H	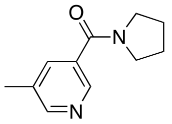	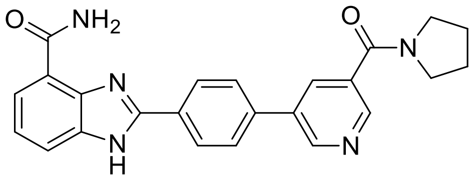
11	UM-C44	425.19	NH_2	CH_3	H	H	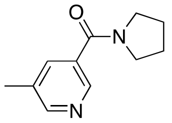	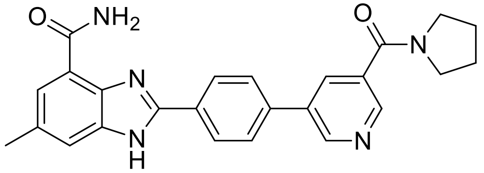
12	UM-C45	411.17	NH_2	H	H	H	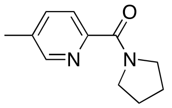	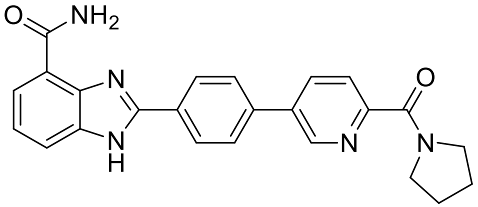
13	UM-C46	425.19	NH_2	CH_3	H	H	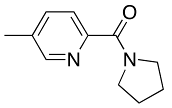	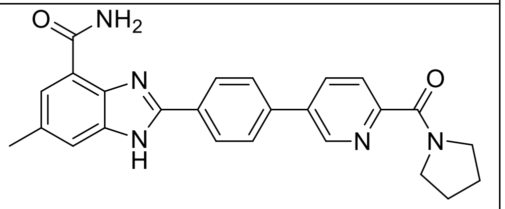
14	UM-C47	314.12	NH_2	H	H	H	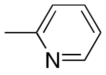	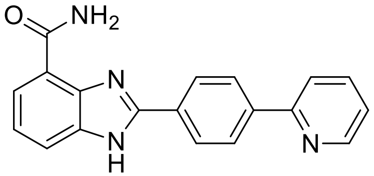

15	UM-C48	370.14	NH_2	H	H	H	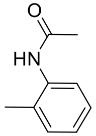	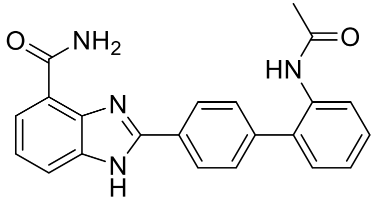
16	UM-C49	370.14	NH_2	H	H	H	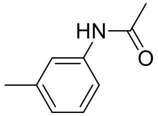	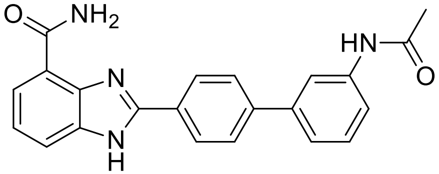
17	UM-C50	370.14	NH_2	H	H	H	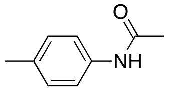	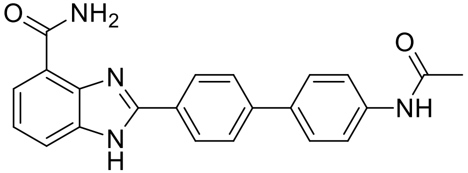
18	UM-C73	410.17	NH_2	H	H	H	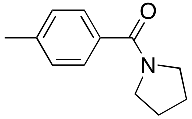	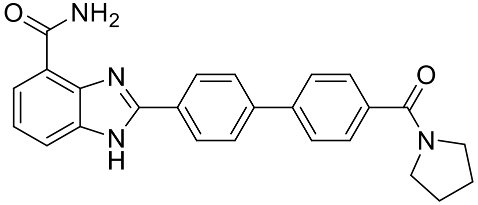
19	UM-C74	424.19	NH_2	CH_3	H	H	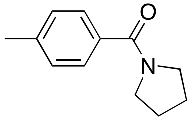	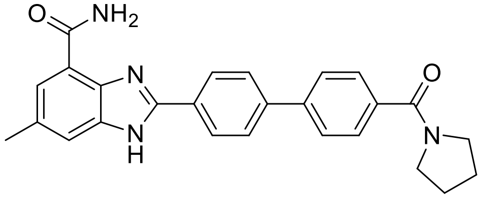
20	UM-C109	371.14	NH_2	H	H	H	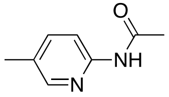	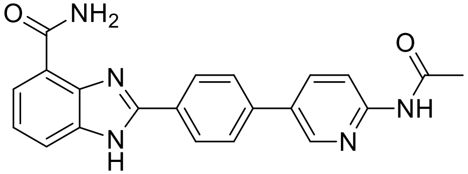

21	UM-C110	397.19	NH_2	H	H	H	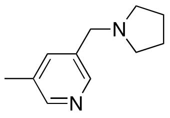	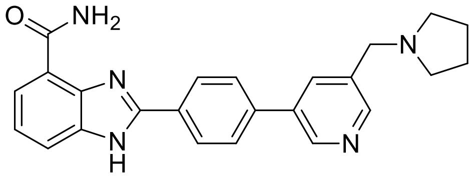
22	UM-C137	385.14	OH	CH_3	H	H	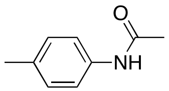	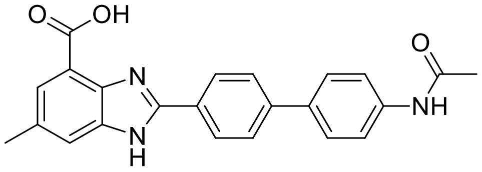
23	UM-C138	385.14	OH	CH_3	H	H	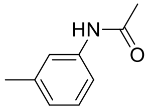	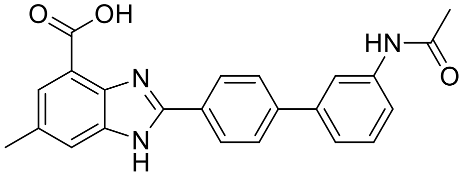
24	UM-C139	385.14	OH	CH_3	H	H	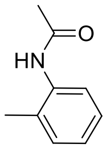	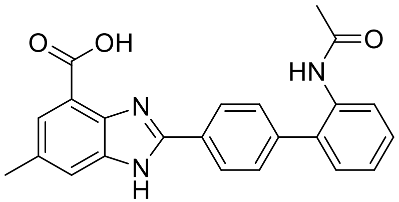
25	UM-C162	491.18	OH	CH_3	H	H	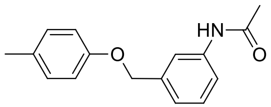	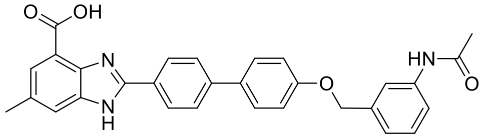
26	UM-C163	385.14	OH	CH_3	H	NHAc	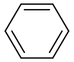	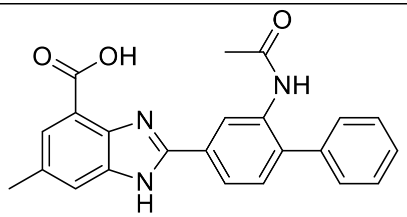

27	UM-C164	454.24	NH_2	H	H	H	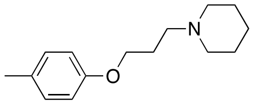	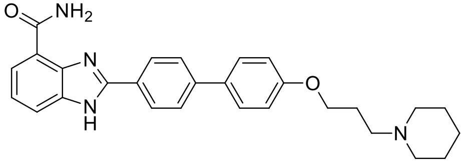
28	UM-C187	491.18	OH	CH_3	H	H	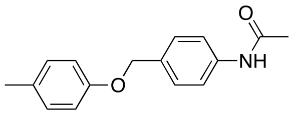	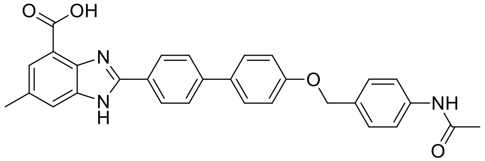
29	UM-C188	491.18	OH	CH_3	H	H	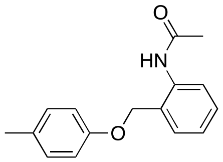	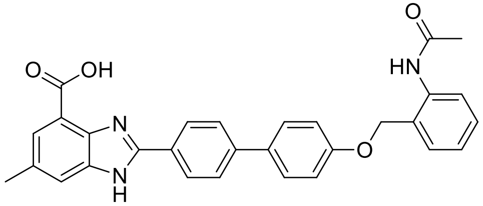
30	UM-C189	440.22	NH_2	H	H	H	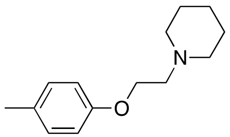	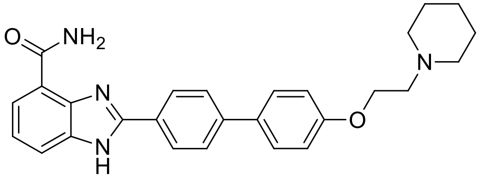
31	UM-C201	450.13	NH_2	F	F	H	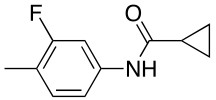	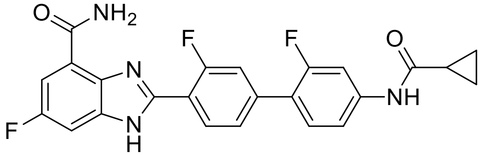
32	UM-C202	402.15	NH_2	F	H	H	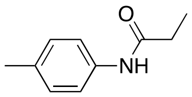	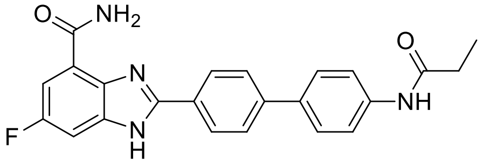

33	UM-C203	432.14	NH_2	F	F	H	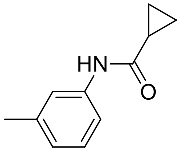	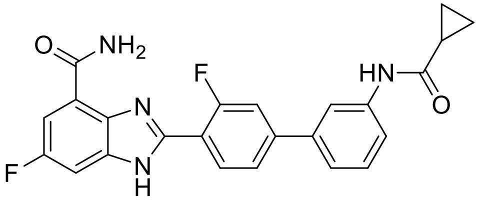
34	UM-C204	462.15	NH_2	F	F	H	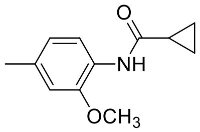	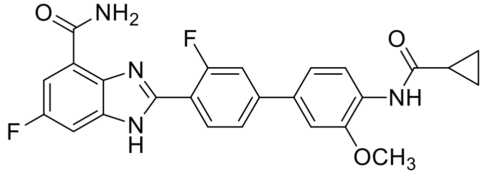
35	UM-C205	444.16	NH_2	F	H	H	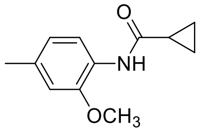	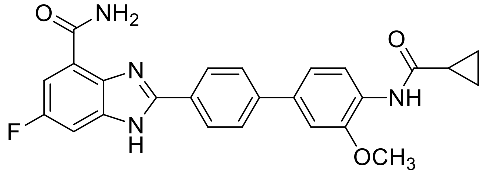

Figure S1.

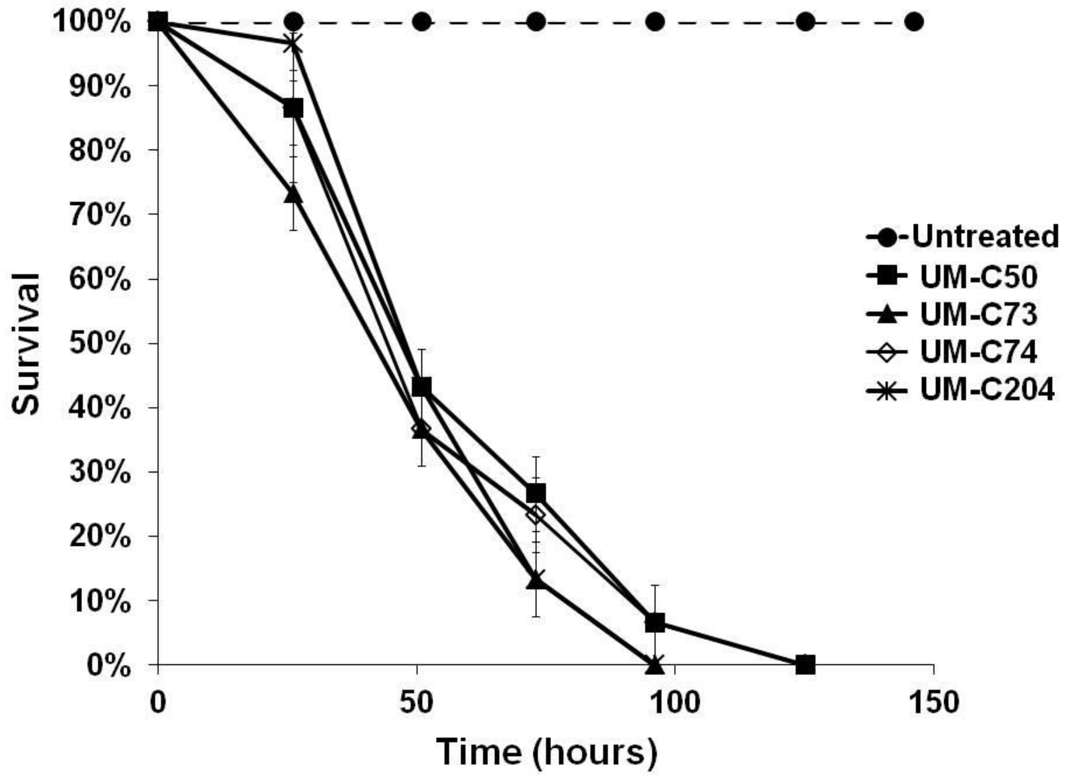

Figure S1. Survival of *C. elegans* fed on heat-killed *E. coli* OP50 in the presence of compounds UM-C50, UM-C73, UM-C74 and UM-C204. Shown is the representative of two independent replicates (n=120). All compounds were tested at 100 µM.

Figure S2.

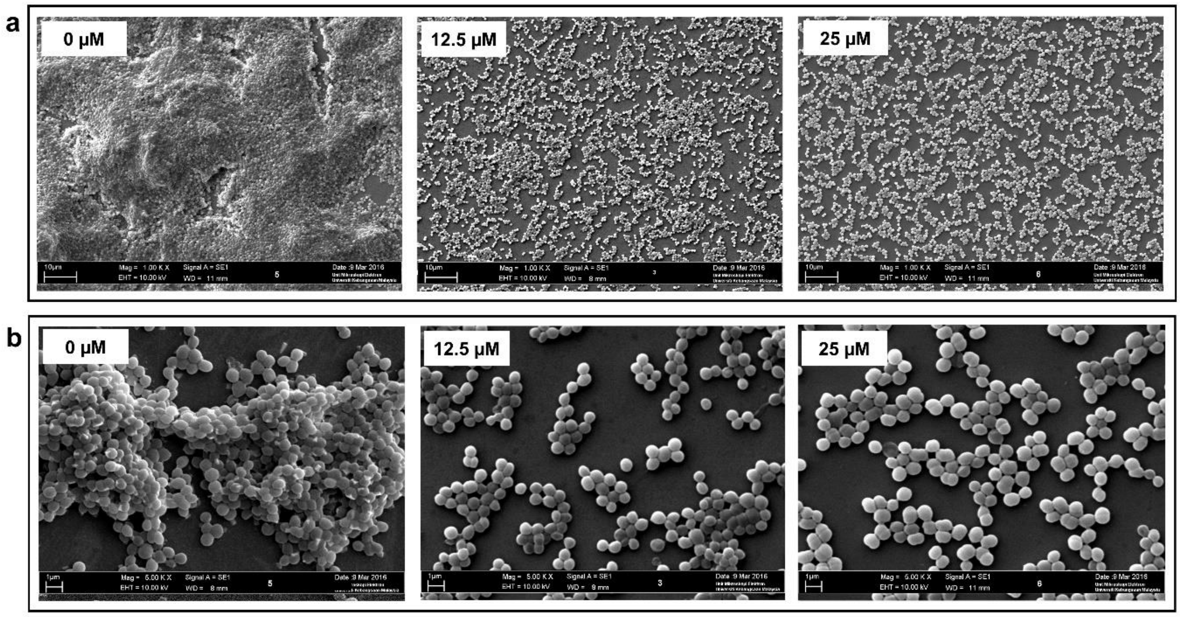

Figure S2. Scanning electron microscopy micrographs of the *S. aureus* biofilm structure at higher concentrations. (**a**) Biofilm formation by untreated (0 µM) and UM-C162-treated *S. aureus* (12.5 µM and 25 µM) following 24 hours incubation (1000x magnification). (b) Bacterial cells cluster in untreated *S. aureus* (0 µM) and the absence of cell aggregates in UM-C162-treated bacteria (12.5 µM and 25 µM) under 5000x magnification.

Figure S3.

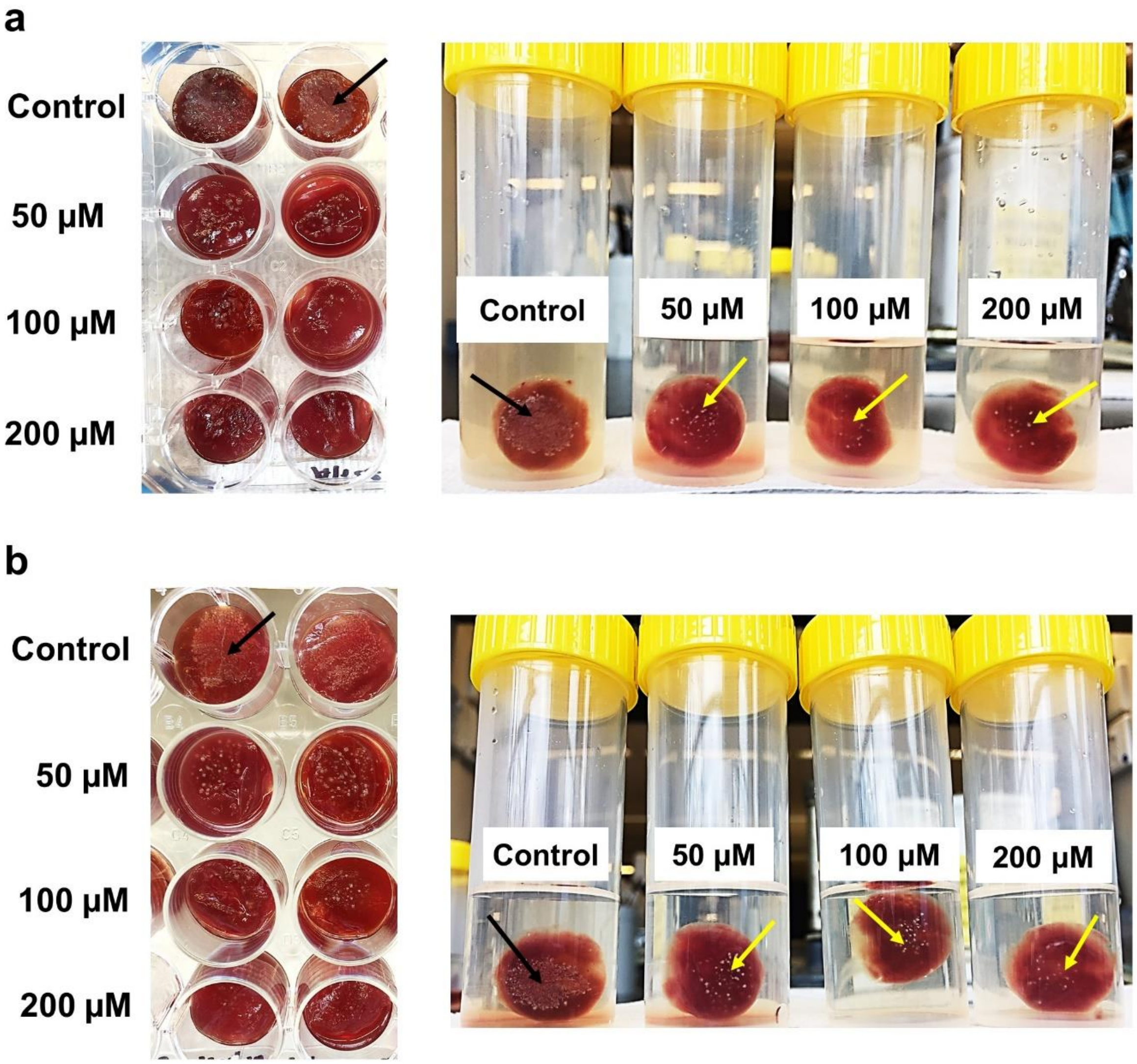

Figure S3. UM-C162 prevents the growth of *S. aureus* and MRSA biofilm in an artificial dermis wound model. Biofilm formation of *S. aureus* (a) and MRSA (b) after exposure to 50 µM, 100 µM and 200 µM of UM-C162. Black arrows indicate the formation of thick biofilm attached to the untreated infected dermis (control) whilst yellow arrows show the absence of biofilm on UM-C162-treated dermis.

Figure S4.

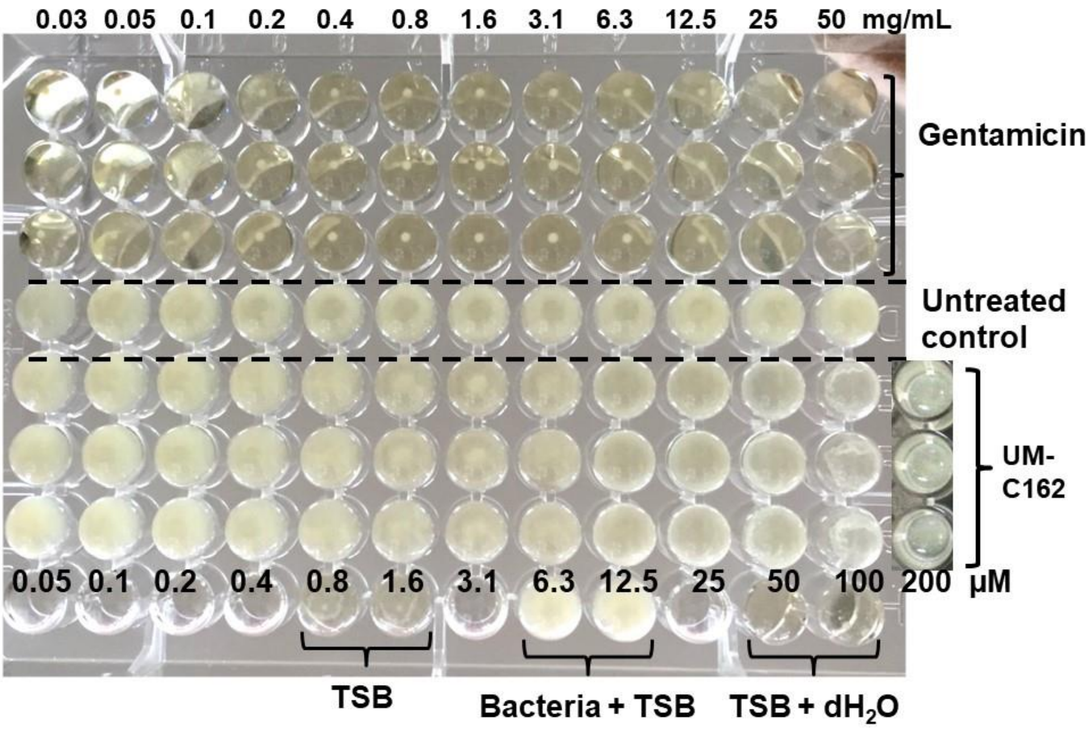

Figure S4. UM-C162 exhibits no antimicrobial effect on *S. aureus*. At UM-C162 concentrations ranging from 0.05 to 200 µM, bacterial growth can be detected in all wells as reflected by the cloudy appearance in the wells. On the other hand, the wells supplemented with the antibiotic gentamicin from 0.03 to 50 mg/mL appear to be clear, indicating a MIC at <30 µg/mL. The untreated control consisting of only the bacteria culture and the dissolving solvent, DMSO, shows apparent bacterial growth whilst the blank controls (TSB only and TSB + dH_2O) are clear after overnight incubation, indicating no bacterial growth.

Table S2: 456 *S. aureus* genes differentially modulated by treatment of UM-C162 as compared to untreated *S. aureus*.

Affymetrix probe ID	ORF	Gene symbol	Description	Fold change	P value
sa_c10648s11101_s_at	SAOUHSC_01311		ABC transporter, ATP-binding protein, putative	294.62	0.000722
sa_c1633s1367_a_at	SAOUHSC_01312		Putative uncharacterized protein	125.66	0.000353
sa_i11106u_x_at	SAOUHSC_01626		Proline dipeptidase, putative	102	0.000038
sa_i3075u_x_at	SAOUHSC_01966		Putative uncharacterized protein	18.68	0.042623
sa_c5329s4605_a_at	SAOUHSC_02646		Putative uncharacterized protein	16.51	0.003238
sa_c5323s4600_a_at	SAOUHSC_02645		Uncharacterized HTH-type transcriptional regulator	15.1	0.004226
sa_c1639s1378_a_at	SAOUHSC_01314		Putative uncharacterized protein	12.71	0.000009
sa_c1635s1372_a_at	SAOUHSC_01313		DNA gyrase B	10.69	0.000209
sa_i11195d_x_at	SAOUHSC_02033		Phage tape measure protein	9.94	0.045438
sa_c10392s10798_at	SAOUHSC_02028		PhiETA ORF57-like protein	8.33	0.027419
sa_i3492ur_x_at	SAOUHSC_01118		Putative uncharacterized protein	8.31	0.035735
sa_c1593s9635_at	SAOUHSC_01296		Putative uncharacterized protein	7.38	0.029201
sa_c3043s2596_a_at	SAOUHSC_00136		Putative uncharacterized protein	6.96	0.024461
sa_c10374s10766_x_at	SAOUHSC_01925		Putative uncharacterized protein	6.72	0.012236
sa_c10704s11153_at	SAV0399		Uncharacterized protein	6.6	0.04078
sa_i3055u_x_at	SAOUHSC_01959		Putative uncharacterized protein	6.58	0.006938
sa_i4210u_x_at	SAOUHSC_02518		Putative uncharacterized protein	6.54	0.049286
sa_c5640s9379_a_at	SAOUHSC_02701		Putative uncharacterized protein	6.53	0.004158
sa_c10269s10678_at		set6	Exotoxin 6	6.46	0.020335
sa_c1561s9623_at	SACOL1586		Uncharacterized protein	6.38	0.036715
sa_c4556s9980_x_at	SAV2151		Uncharacterized protein	6.29	0.040922
sa_c6443s5614_a_at	SAOUHSC_02943		Citrate transporter, putative	6.27	0.019834
sa_c10708s11157_at	SAV0403		Uncharacterized protein	6.19	0.000759
sa_c192s9522_a_at	SA0055		Uncharacterized protein	5.98	0.002413
sa_c9963s8658_a_at	SACOL2240	rpsJ	30S ribosomal protein S10	5.97	0.004152

sa_c7338s6377_a_at	SAOUHSC_00412		NADH dehydrogenase subunit 5, putative	5.93	0.00556
sa_c4852s4158_at	SAOUHSC_02501	rplX	50S ribosomal protein L24	5.71	0.004857
sa_c740s548_a_at	SAOUHSC_01032		Cytochrome d ubiquinol oxidase, subunit II, putative	5.68	0.002142
sa_c6929s10116_at	SAOUHSC_00282		Branched-chain amino acid transport system carrier protein	5.59	0.024382
sa_c10713s11162_a_at	SAV0409		Similar to DNA translocase FtsK	5.55	0.006045
sa_c2333s9718_x_at	SAOUHSC_01544		Hypothetical phage protein	5.31	0.044745
sa_i7519ur_x_at	SAOUHSC_00857		Putative uncharacterized protein	5.26	0.048948
sa_c1603s9640_x_at	SAOUHSC_01297		Putative uncharacterized protein	5.13	0.000505
sa_c3941s3408_at	SAOUHSC_02156		Putative uncharacterized protein	5.11	0.002968
sa_c6147s5329_at	SAOUHSC_02853		Putative uncharacterized protein	5.11	0.035804
sa_c5600s4854_a_at	SAOUHSC_02688		Putative uncharacterized protein	5.08	0.026249
sa_c4836s4142_at	SAOUHSC_02495	rplR	50S ribosomal protein L18	4.97	0.010388
sa_i2904u_x_at	SAOUHSC_01883		Putative uncharacterized protein	4.96	0.000097
sa_c2719s2293_at	SAOUHSC_01689	rpsT	30S ribosomal protein S20	4.82	0.001799
sa_c4860s4166_at	SAOUHSC_02504	rpmC	50S ribosomal protein L29	4.77	0.003248
sa_c7344s6381_a_at	SAOUHSC_00413		UPF0753 protein	4.74	0.009339
sa_c1687s1419cv_at	SAOUHSC_01651	rpmG2	50S ribosomal protein L33 2	4.71	0.017634
sa_c9420s8234_a_at	SAOUHSC_02932	betA	Oxygen-dependent choline dehydrogenase	4.69	0.044798
sa_c9925s10469_a_at	SAOUHSC_02248		Putative uncharacterized protein	4.68	0.003941
sa_c6694s5837_a_at	SAOUHSC_03006.1		Putative uncharacterized protein	4.51	0.001026
sa_c470s302_at	SAOUHSC_00957		Putative uncharacterized protein	4.43	0.019245
sa_c10191s8871_a_at	SAOUHSC_02503	rpsQ	30S ribosomal protein S17	4.36	0.023101
sa_c3556s9823_at		sen	Enterotoxin	4.26	0.004393
sa_c736s544_a_at	SAOUHSC_01031		Cytochrome d ubiquinol oxidase, subunit I, putative	4.22	0.015548
sa_c4515s3860_a_at	SAOUHSC_02382		Putative uncharacterized protein	4.17	0.000753
sa_c2144s1843_a_at	SAOUHSC_01468		Putative uncharacterized protein	4.16	0.001124
sa_c8637s7585_a_at	SAOUHSC_00881		Putative uncharacterized protein	4.11	0.001074

sa_c1057s841_a_at	SAOUHSC_01134		Putative uncharacterized protein	4.08	0.014532
sa_i3133ur_x_at	SAOUHSC_00154		Putative uncharacterized protein	4.07	0.009722
sa_c4608s3937_a_at	SAOUHSC_02419	sepA	Multidrug resistance efflux pump	4.01	0.001444
sa_c1551s9617_at	SACOL1577		Uncharacterized protein	3.98	0.013428
sa_c3487s9161_a_at	SAOUHSC_01921		Putative uncharacterized protein	3.96	0.020848
sa_c4005s9893_a_at	SA1769		Uncharacterized protein	3.96	0.002281
sa_i9270d_x_at	SA2282		Uncharacterized protein	3.93	0.02794
sa_c582s408_a_at	SAOUHSC_00991		Putative uncharacterized protein	3.91	0.000549
sa_c10524s10977cv_s_at	SAOUHSC_02838		Putative uncharacterized protein	3.88	0.047264
sa_c10125s8848_a_at	SAOUHSC_02157		Putative uncharacterized protein	3.8	0.032109
sa_i10370ur_x_at	SAOUHSC_03008		Imidazole glycerol phosphate synthase subunit hisF	3.76	0.014648
sa_c10456s10893_a_at	SAOUHSC_02206		Hypothetical phage protein	3.75	0.03835
sa_c4081s3441_a_at	SAOUHSC_00171		Gamma-glutamyltranspeptidase, putative	3.7	0.013464
sa_c9067s7955_a_at	SAOUHSC_00965		Putative uncharacterized protein	3.69	0.000307
sa_c9955s8651_a_at	SAOUHSC_02502	rplN	50S ribosomal protein L14	3.68	0.005626
sa_c4876s4184_at	SAOUHSC_02508	rpsS	30S ribosomal protein S19	3.63	0.005576
sa_c4880s4187_at	SAOUHSC_02511	rplD	50S ribosomal protein L4	3.61	0.024593
sa_c1175s958_a_at	SAOUHSC_01173		Putative uncharacterized protein	3.6	0.003386
sa_c4868s4175_a_at	SAOUHSC_02506	rpsC	30S ribosomal protein S3	3.58	0.00532
sa_c4888s4195_a_at	SAOUHSC_02512	rplC	50S ribosomal protein L3	3.53	0.04621
sa_c3294s2837_at	SAOUHSC_01863		Putative uncharacterized protein	3.5	0.010165
sa_c4844s4152_at	SAOUHSC_02499	rpsZ; rpsN1	30S ribosomal protein S14 type Z	3.5	0.005675
sa_c10228s8907_a_at	SAOUHSC_02794		Putative uncharacterized protein	3.48	0.000348
sa_c8951s10282_a_at	SAOUHSC_01926		Putative uncharacterized protein	3.48	0.014332
sa_c10598s11052_s_at	SAOUHSC_02821		Membrane spanning protein, putative	3.4	0.000838
sa_c10192s8875_a_at	SAOUHSC_02510	rplW	50S ribosomal protein L23	3.38	0.005162
sa_c4840s4147_a_at	SAOUHSC_02498	rpsH	30S ribosomal protein S8	3.38	0.005302
sa_c7621s6634_a_at	SAOUHSC_00520	rplJ	50S ribosomal protein L10	3.38	0.009176
sa_c10325s10715cv_s_a	SACOL1839		Transposase, IS200 family	3.37	0.016459

t

sa_c6934s6055_a_at	SAOUHSC_00284		5'-nucleotidase, lipoprotein e(P4) family	3.36	0.011279
sa_c4872s4181_at	SAOUHSC_02507	rplV	50S ribosomal protein L22	3.32	0.006044
sa_c5972s5179_a_at	SAOUHSC_02795		Putative uncharacterized protein	3.31	0.000386
sa_i10774u_x_at	SAOUHSC_00194		Putative uncharacterized protein	3.28	0.00091
sa_c1398s1173_at	SAOUHSC_01247	rbfA	Ribosome-binding factor A	3.27	0.004177
sa_c9951s8647_at	SAOUHSC_02496	rplF	50S ribosomal protein L6	3.18	0.009184
sa_c1431s1205_a_at	SAOUHSC_01256		Putative uncharacterized protein	3.15	0.002842
sa_c3188s2737_a_at	SAOUHSC_01829	rpsD	30S ribosomal protein S4	3.1	0.010409
sa_c3623s3103_a_at	SAOUHSC_01979		Putative uncharacterized protein	3.1	0.000122
sa_c9601s8362_a_at	SAOUHSC_01254		Putative uncharacterized protein	3.07	0.006959
sa_c1723s1460_a_at	SAOUHSC_01340		Putative uncharacterized protein	3.06	0.013175
sa_c6495s5664_a_at	SAOUHSC_02960		Putative uncharacterized protein	3.04	0.017707
sa_c9605s8367_a_at	SAOUHSC_01238		Phosphatidate cytidylyltransferase	3.04	0.001187
sa_c493s322_at	SAOUHSC_00964		Putative uncharacterized protein	3.03	0.003255
sa_c537s363_a_at	SAOUHSC_00978		Putative uncharacterized protein	3.02	0.001623
sa_c197s9528_at	SACOL0040		Uncharacterized protein	3	0.039444
sa_c1264s1041_at	SAOUHSC_01201	acpP; hmrB	Acyl carrier protein	2.98	0.043614
sa_c7365s6401_a_at	SAOUHSC_00420		Transporter	2.98	0.002373
sa_c9241s8090_a_at	SAOUHSC_00182		Putative uncharacterized protein	2.98	0.007723
sa_c7832s10211_a_at	SAOUHSC_00584		Putative uncharacterized protein	2.97	0.029183
sa_c3428s2956_a_at	SAOUHSC_01902		Putative uncharacterized protein	2.96	0.001987
sa_c7070s6181_a_at	SAOUHSC_00330		Putative uncharacterized protein	2.95	0.038993
sa_i2636ur_x_at	SAOUHSC_01800.1		Putative uncharacterized protein	2.95	0.015923
sa_c1711s1447_a_at	SAOUHSC_01338		UPF0154 protein	2.94	0.003616
sa_c4864s4170_at	SAOUHSC_02505	rplP	50S ribosomal protein L16	2.94	0.014236
sa_c2369s9745_at	SAOUHSC_01556		PVL orf 52-like protein	2.93	0.022118
sa_c813s612_a_at	SAOUHSC_01051		Putative uncharacterized protein	2.93	0.001716
sa_i2573u_x_at	SAOUHSC_01780		Putative uncharacterized protein	2.93	0.00115
sa_c1960s1685_a_at	SACOL1438		Uncharacterized protein	2.92	0.003517

sa_c489s318_a_at	SAOUHSC_00963		Lipoate-protein ligase	2.9	0.001119
sa_i7154ur_x_at	SAOUHSC_00709		Putative uncharacterized protein	2.9	0.025774
sa_c152s9488_at		ermA	rRNA adenine N-6-methyltransferase	2.88	0.044664
sa_c6760s5903_a_at	SAOUHSC_03023	drp35	Lactonase	2.86	0.003577
sa_c9959s8654_a_at	SAOUHSC_02509	rplB	50S ribosomal protein L2	2.85	0.023338
sa_i6279u_x_at	SAOUHSC_00358.1		Putative uncharacterized protein	2.84	0.030237
sa_c8481s7443_a_at	SAOUHSC_00830		Putative uncharacterized protein	2.83	0.025779
sa_c10078s8796_a_at	SAOUHSC_01739	lytH	Probable cell wall amidase	2.82	0.003363
sa_c2214s1915_at	SAOUHSC_01490		DNA-binding protein HU, putative	2.81	0.011536
sa_c4848s4156_at	SAOUHSC_02500	rplE	50S ribosomal protein L5	2.8	0.028964
sa_c4832s4138_at	SAOUHSC_02494	rpsE	30S ribosomal protein S5	2.77	0.023489
sa_c10288s8968_a_at	SAOUHSC_00775		Putative uncharacterized protein	2.76	0.009737
sa_i2428ur_x_at	SAOUHSC_01736		Putative uncharacterized protein	2.76	0.029721
sa_c1810s1538_a_at	SAOUHSC_01364	tyrA	Prephenate dehydrogenase	2.72	0.003025
sa_c8633s7584_a_at	SAOUHSC_00880		Putative uncharacterized protein	2.72	0.042562
sa_c2564s2141_a_at	SAOUHSC_01632	gcvPB	Glycine decarboxylase subunit 2	2.71	0.00523
sa_c3774s3246_a_at	SAOUHSC_02110		Putative uncharacterized protein	2.71	0.000384
sa_c3992s9884_a_at	SA1766		Uncharacterized protein	2.7	0.044601
sa_c8768s7712_at	SACOL1136		Uncharacterized protein	2.69	0.00416
sa_c1449s1223_a_at	SAOUHSC_01264		Putative uncharacterized protein	2.68	0.008205
sa_c2875s2441_a_at	SAOUHSC_01741	dtd	D-aminoacyl-tRNA deacylase	2.66	0.004239
sa_c525s350_at	SAOUHSC_00975		Putative uncharacterized protein	2.65	0.017037
sa_c925s720_a_at	SAOUHSC_01089	isdG	Heme oxygenase (staphylobilin-producing) 1	2.59	0.001291
sa_i3869u2_x_at	SAOUHSC_02385		Mannose-6-phosphate isomerase, class I	2.57	0.042354
sa_c10171s8867_at	SAOUHSC_02384		Putative uncharacterized protein	2.56	0.014691
sa_c2572s2149_a_at	SAOUHSC_01634	gcvT	Aminomethyltransferase	2.56	0.021299
sa_c5592s4849_a_at	SAOUHSC_02686		Putative uncharacterized protein	2.56	0.002676
sa_i10628dr_x_at	SAOUHSC_02521		Putative uncharacterized protein	2.55	0.048991
sa_c6673s5826_a_at	SAOUHSC_03001	icaR	Biofilm operon icaADBC HTH-type negative transcriptional regulator	2.54	0.002358

sa_c1132s914_a_at	SAOUHSC_01160		Putative uncharacterized protein	2.52	0.003649
sa_c1230s1008_at	SAOUHSC_00074		Periplasmic binding protein, putative	2.52	0.031299
sa_c10498s10949_s_at	SAOUHSC_02555		Putative uncharacterized protein	2.5	0.006523
sa_c9611s8371_a_at	SAOUHSC_01237		Isoprenyl transferase	2.49	0.001046
sa_c5885s5122_a_at	SAOUHSC_02777		Putative uncharacterized protein	2.46	0.015485
sa_c8117s7099_a_at	SAOUHSC_00691	uppP	Undecaprenyl-diphosphatase	2.46	0.030884
sa_c8497s7459_a_at	SAOUHSC_00834		Thioredoxin, putative	2.45	0.003189
sa_c2392s9756_a_at	SAOUHSC_01570		PVL orf 37-like protein	2.44	0.040032
sa_c3639s3120_a_at	SAOUHSC_01984		Putative uncharacterized protein	2.44	0.003728
sa_c7236s6302_a_at	SAOUHSC_00367		Putative uncharacterized protein	2.43	0.018963
sa_c5696s9156_a_at	SAOUHSC_02716	bioD	ATP-dependent dethiobiotin synthetase	2.42	0.000288
sa_c9309s8152_at	SAOUHSC_02596		Putative uncharacterized protein	2.42	0.00386
sa_c9927s10470_a_at	SAOUHSC_02249		Putative uncharacterized protein	2.41	0.002343
sa_c5823s5065_a_at	SAOUHSC_02761		Putative uncharacterized protein	2.38	0.007958
sa_c10576s9058_a_at	SAOUHSC_01038	def	Peptide deformylase	2.37	0.001112
sa_c546s374_a_at	SAOUHSC_00980	menA	1,4-dihydroxy-2-naphthoate octaprenyltransferase	2.37	0.004814
sa_c4892s4201_a_at	SAOUHSC_02515		Putative uncharacterized protein	2.34	0.000013
sa_c5299s4579_a_at	SAOUHSC_02638		Putative uncharacterized protein	2.34	0.003819
sa_c6967s6091_a_at	SAOUHSC_00297		Putative uncharacterized protein	2.33	0.000066
sa_c1785s1516_a_at	SAOUHSC_01357		Putative uncharacterized protein	2.32	0.001431
sa_c3696s3175_a_at	SACOL1928		Uncharacterized protein	2.32	0.000263
sa_c6029s5227_a_at	SAOUHSC_00431		Putative uncharacterized protein	2.32	0.014187
sa_c791s594_at	SAOUHSC_01045		Putative uncharacterized protein	2.32	0.005673
sa_c8147s7131_a_at	SACOL0752		Uncharacterized protein	2.32	0.019977
sa_c8176s7154_a_at	SAOUHSC_00710		N-acetylglucosamine-6-phosphate deacetylase	2.32	0.011343
sa_c4985s4289_a_at	SAOUHSC_00200		Membrane protein, putative	2.31	0.000804
sa_c10520s10969_at	SAOUHSC_02818		Putative uncharacterized protein	2.3	0.044237
sa_c4365s3717_a_at	SAOUHSC_02327	yidC	Membrane protein insertase YidC	2.3	0.016943
sa_c4530s3875_a_at	SAOUHSC_02388		Putative uncharacterized protein	2.3	0.002931
sa_c9750s10427_at	SAOUHSC_01131		Putative uncharacterized protein	2.3	0.004642

AFFX-TransRecMur/X57349_3_at		Tfrc	Transferrin receptor protein 1	2.29	0.031904
sa_c8053s7040_a_at	SAOUHSC_00673		Putative uncharacterized protein	2.29	0.001541
sa_c8734s7676_a_at	SAOUHSC_01788	thrS	Threonine--tRNA ligase	2.28	0.002931
sa_c10246s10660_at	SAOUHSC_02927	mqo	Probable malate:quinone oxidoreductase	2.27	0.026274
sa_c7841s6831_a_at	SAOUHSC_00603		Putative uncharacterized protein	2.27	0.004131
sa_i9235d2_x_at	SAOUHSC_02411		Putative uncharacterized protein	2.27	0.036463
sa_c1536s1314_a_at	SAOUHSC_01287		Glutamine synthetase	2.25	0.046298
sa_c933s729_a_at	SAOUHSC_01093	pheT	Phenylalanine--tRNA ligase beta subunit	2.25	0.001065
sa_c4792s4098_at	SAOUHSC_02484	rplQ	50S ribosomal protein L17	2.24	0.003423
sa_c10203s8884_a_at	SAOUHSC_02592		Putative uncharacterized protein	2.23	0.002042
sa_c4651s3971cs_s_at	SAOUHSC_02433		Putative uncharacterized protein	2.23	0.001226
sa_c6391s5562_a_at	SAOUHSC_02926	fda	Fructose-bisphosphate aldolase class 1	2.23	0.041996
sa_i285u_x_at	SAOUHSC_00952	ltaA	Probable glycolipid permease	2.23	0.038463
sa_c7681s6690_a_at	SAOUHSC_00540		Putative uncharacterized protein	2.21	0.023029
sa_c4482s3827_a_at	SAOUHSC_02368	pyrG	CTP synthase	2.2	0.041235
sa_c5606s4859_a_at	SAOUHSC_02689		Putative uncharacterized protein	2.2	0.005181
sa_c3615s3095_a_at	SAOUHSC_01977		UPF0342 protein	2.19	0.006068
sa_c5349s4625_a_at	SAOUHSC_01046	potA	Spermidine/putrescine import ATP-binding protein	2.19	0.018177
sa_c8103s7087_a_at	SAOUHSC_00687		Putative uncharacterized protein	2.19	0.000487
sa_c8772s7713_a_at	SAOUHSC_01075	coaD	Phosphopantetheine adenylyltransferase	2.19	0.00152
sa_c8842s7778_a_at	SAOUHSC_00539		Putative uncharacterized protein	2.19	0.003585
sa_c2570s2145_a_at	SAOUHSC_01633	gcvPA	Probable glycine dehydrogenase (decarboxylating) subunit 1	2.18	0.001726
sa_c5439s4708_a_at	SAOUHSC_00693		Putative uncharacterized protein	2.18	0.019301
sa_c5274s4572_a_at	SAOUHSC_02630		Putative uncharacterized protein	2.17	0.0378
sa_c342s182_a_at	SAOUHSC_02629		Drug resistance transporter	2.16	0.019573
sa_c5507s10021cs_s_at	SAOUHSC_02663		Putative uncharacterized protein	2.16	0.004085
sa_c6877s10101_at	SA0553		Uncharacterized protein	2.16	0.038924
sa_c8005s6991_at	SAOUHSC_00658		Putative uncharacterized protein	2.16	0.018615
sa_c1643s1381_at	SAOUHSC_01316		Putative uncharacterized protein	2.15	0.022016

sa_c4828s4134_x_at	SAOUHSC_02493	rpmD	50S ribosomal protein L30	2.15	0.014046
sa_c8552s7512_a_at	SAOUHSC_00855		Probable nitronate monooxygenase	2.15	0.008901
sa_c3028s2585_at	SAOUHSC_01784	rplT	50S ribosomal protein L20	2.14	0.020854
sa_c10215s10637_s_at	SAOUHSC_02729		Amino acid ABC transporter-like protein, putative	2.13	0.003487
sa_c1478s1255_a_at	SAOUHSC_01272	mutS	DNA mismatch repair protein	2.13	0.002966
sa_c1974s1697_a_at	SAOUHSC_01411		Putative branched-chain amino acid carrier protein	2.13	0.004378
sa_c4498s3842_a_at	SAOUHSC_02376		Putative uncharacterized protein	2.13	0.035334
sa_c8256s7233_at	SAOUHSC_00739	queF	NADPH-dependent 7-cyano-7-deazaguanine reductase	2.13	0.018593
sa_i1041u_x_at	SAOUHSC_01200		Putative uncharacterized protein	2.13	0.021208
sa_i3597ur_x_at	SAOUHSC_02296		Protein SprT-like	2.13	0.034443
sa_c1579s9626_a_at	SAOUHSC_02016		Conserved hypothetical phage protein	2.11	0.00755
sa_c2922s2484_a_at	SAOUHSC_01755	rpmA	50S ribosomal protein L27	2.11	0.003632
sa_c3361s2898_a_at	SAOUHSC_01878		Putative uncharacterized protein	2.11	0.021766
sa_c5676s4926_a_at	SAOUHSC_02712	bioW	6-carboxyhexanoate--CoA ligase	2.11	0.030272
sa_c9334s8169_a_at	SAOUHSC_02668		Putative uncharacterized protein	2.11	0.000937
sa_c10366s10758cv_s_at	SAOUHSC_01732		Putative uncharacterized protein	2.1	0.024465
sa_c8502s10249cs_s_at	SAOUHSC_00838		Putative uncharacterized protein	2.1	0.049511
sa_c1620s1365_a_at	SAOUHSC_01309		Putative uncharacterized protein	2.09	0.025396
sa_c5680s4930_a_at	SAOUHSC_02713		Putative uncharacterized protein	2.09	0.040311
sa_c4034s3428_a_at	SAOUHSC_00169		Peptide ABC transporter, permease protein, putative	2.08	0.020658
sa_c1425s1202_a_at	SAOUHSC_01255		Putative uncharacterized protein	2.07	0.002649
sa_c3186s2733_a_at	SAOUHSC_01828		GAF domain protein	2.07	0.016942
sa_c6333s5501_a_at	SAOUHSC_02910		Putative uncharacterized protein	2.07	0.039699
sa_c9811s8552_a_at	SAOUHSC_02369	rpoE	Probable DNA-directed RNA polymerase subunit delta	2.06	0.012086
sa_c1494s1273_at	SAOUHSC_01275		Putative uncharacterized protein	2.04	0.027575
sa_c3507s3024_a_at	SAOUHSC_00398		Restriction modification system specificity subunit,	2.04	0.04971

			putative		
sa_c4239s3588_a_at	SAOUHSC_02288	leuD	3-isopropylmalate dehydratase small subunit	2.04	0.03028
sa_c9882s8618_a_at	SAOUHSC_02125		Putative uncharacterized protein	2.04	0.017615
sa_c2487s2066_a_at	SACOL1557		Uncharacterized protein	2.03	0.006422
sa_c7851s6843_a_at	SAOUHSC_00607		Putative uncharacterized protein	2.03	0.022458
sa_c9653s8413_a_at	SAOUHSC_01666	glyQS	Glycine--tRNA ligase	2.03	0.014401
sa_c10061s8767_at	SAOUHSC_01406	acyP	Acylphosphatase	2.02	0.001378
sa_c5081s4376_a_at	SAOUHSC_02575		Putative uncharacterized protein	2.02	0.011947
sa_c6044s5243_a_at	SAOUHSC_02824		Putative uncharacterized protein	2.02	0.018407
sa_c1739s1473_a_at	SAOUHSC_01344		Putative uncharacterized protein	2.01	0.013253
sa_c9141s8010_at	SAOUHSC_01971		Putative uncharacterized protein	2.01	0.027184
sa_i4391u_x_at	SAOUHSC_02580.1		Putative uncharacterized protein	2.01	0.00136
sa_c5788s5026_a_at	SAOUHSC_02747		Putative uncharacterized protein	-2.01	0.016371
sa_i2813d_x_at	SAOUHSC_01854		Putative uncharacterized protein	-2.01	0.035666
sa_c3728s3205_a_at	SAOUHSC_02097		Putative uncharacterized protein	-2.02	0.000237
sa_c1994s9145_a_at	SAOUHSC_01418	odhA	2-oxoglutarate dehydrogenase E1 component	-2.03	0.001392
sa_c3399s2929_a_at	SAOUHSC_01890		Putative uncharacterized protein	-2.03	0.017817
sa_c3850s3320_a_at	SAOUHSC_00158		PTS system EIIBC component	-2.03	0.007899
sa_c9989s8682_a_at	SAOUHSC_01172	pyrE	Orotate phosphoribosyltransferase	-2.03	0.031617
sa_i10245u_x_at	SACOL0865		Uncharacterized protein	-2.03	0.001526
sa_c7199s6265_a_at	SAOUHSC_00356		Putative uncharacterized protein	-2.04	0.025984
sa_c3422s2952_a_at	SAOUHSC_01900		Putative uncharacterized protein	-2.05	0.005939
sa_c7931s6914_a_at	SAOUHSC_00628	mnhD2; mrpD2	Putative antiporter subunit	-2.05	0.017194
sa_c965s755_at	SAOUHSC_01103		Succinate dehydrogenase cytochrome b-558 subunit, putative	-2.05	0.002805
sa_c3270s2813_at	SAOUHSC_01855		UPF0478 protein	-2.06	0.00502
sa_i4014ur_x_at	SAOUHSC_00189		Putative uncharacterized protein	-2.06	0.018394
sa_c1458s1237_a_at	SAOUHSC_01267		Putative uncharacterized protein	-2.07	0.042416
sa_c1825s9344_a_at	SAOUHSC_00094		Uncharacterized protein	-2.07	0.016634

sa_c5560s4814_a_at	SAOUHSC_02678		Respiratory nitrate reductase, gamma subunit	-2.07	0.006821
sa_c570s397_a_at	SAOUHSC_00988	sspA	Glutamyl endopeptidase	-2.07	0.01769
sa_c8990s7897_at	SAOUHSC_00799	eno	Enolase	-2.07	0.007158
sa_c5516s4772_a_at	SAOUHSC_02665		Putative uncharacterized protein	-2.08	0.023831
sa_c8043s7027_a_at	SAOUHSC_00668		ABC transporter permease, putative	-2.08	0.000887
sa_c904s700_a_at	SAOUHSC_00060		Putative uncharacterized protein	-2.08	0.003651
sa_c9253s8099_a_at	SAOUHSC_00227		Putative uncharacterized protein	-2.08	0.01711
sa_c10218s8900_a_at	SAOUHSC_02757		Antitoxin	-2.09	0.007769
sa_c10293s8975_at	SAOUHSC_00807		Putative uncharacterized protein	-2.1	0.014604
sa_c6299s5473_a_at	SAOUHSC_02900		Uncharacterized hydrolase	-2.1	0.009114
sa_c3643s3123_a_at	SAOUHSC_01987		Putative uncharacterized protein	-2.12	0.001848
sa_c7633s6648_a_at	SAOUHSC_00524	rpoB	DNA-directed RNA polymerase subunit beta	-2.12	0.000143
sa_c1045s829_a_at	SAOUHSC_01127		Putative uncharacterized protein	-2.13	0.029903
sa_c2417s9774cv_s_at	SAOUHSC_01583		Conserved hypothetical phage protein	-2.13	0.038051
sa_c8424s7394_a_at	SAOUHSC_00808		Putative uncharacterized protein	-2.13	0.021525
sa_c7883s6875_a_at	SAOUHSC_00616		Putative uncharacterized protein	-2.14	0.023138
sa_c1s2_a_at	SAOUHSC_00001	dnaA	Chromosomal replication initiator protein	-2.15	0.002101
sa_c10627s11083cv_s_at	SAOUHSC_00875		Putative uncharacterized protein	-2.16	0.017605
sa_c7431s6453_a_at	SAOUHSC_00441		Putative uncharacterized protein	-2.16	0.004797
sa_c7637s6652_a_at	SAOUHSC_00525	rpoC	DNA-directed RNA polymerase subunit beta	-2.16	0.003953
sa_c8934s7849_a_at	SAOUHSC_00716		Putative uncharacterized protein	-2.17	0.00507
sa_c10526s10981_a_at	SAOUHSC_02857		Putative uncharacterized protein	-2.19	0.044782
sa_c10546s11006_s_at	SAOUHSC_00776		Excinuclease ABC, B subunit	-2.19	0.006897
sa_c6341s5509_a_at	SAOUHSC_00234		Putative uncharacterized protein	-2.19	0.009987
sa_c4755s4067_a_at	SAOUHSC_02466		Truncated MHC class II analog protein	-2.2	0.007057
sa_c10365s10757cv_s_at	SAOUHSC_01725	mnmA	tRNA-specific 2-thiouridylase	-2.21	0.014906
sa_c1457s1233_a_at	SAOUHSC_01266		Putative uncharacterized protein	-2.21	0.000987
sa_c6411s5581_a_at	SAOUHSC_00236		6-phospho-beta-glucosidase, putative	-2.21	0.002044

sa_c7224s6289_a_at	SAOUHSC_00364	ahpF	Alkyl hydroperoxide reductase subunit F	-2.22	0.043294
sa_c10684s11134cv_s_at	SAOUHSC_00183		Membrane protein, putative	-2.23	0.004424
sa_c4971s4276_a_at	SAOUHSC_02543	moaC	Cyclic pyranopterin monophosphate synthase accessory protein	-2.24	0.006827
sa_c6506s5675_a_at	SAOUHSC_02963	clfB	Clumping factor B	-2.25	0.001858
sa_c9495s8286_a_at	SAOUHSC_00037		Putative uncharacterized protein	-2.26	0.025397
sa_c3979s9871_at	SA1762		Uncharacterized protein	-2.27	0.006279
sa_c4038s9918_s_at	SAOUHSC_00383		Putative uncharacterized protein	-2.27	0.000898
sa_c2851s2417_at	SAOUHSC_01729		Putative uncharacterized protein	-2.29	0.026492
sa_c9344s8176_a_at	SAOUHSC_02724		Putative uncharacterized protein	-2.29	0.00353
sa_c8485s7447_a_at	SAOUHSC_00831		Organic hydroperoxide resistance protein-like	-2.3	0.010937
sa_c3951s9849_a_at	SAOUHSC_02163	hlb	Phospholipase C	-2.31	0.011288
sa_c6086s5272_a_at	SAOUHSC_00228		Putative uncharacterized protein	-2.31	0.028357
sa_c1996s1713_a_at	SAOUHSC_01419	arlS	Signal transduction histidine-protein kinase	-2.32	0.014091
sa_c3976s9867_at	SAOUHSC_00163		Putative uncharacterized protein	-2.32	0.02062
sa_c5855s5097_a_at	SAOUHSC_02769		Putative uncharacterized protein	-2.32	0.040251
sa_c10217s8896_a_at	SAOUHSC_02756		Putative uncharacterized protein	-2.33	0.010118
sa_c3053s2604_a_at	SAOUHSC_01792		Putative uncharacterized protein	-2.34	0.001672
sa_c3870s3341_a_at	SAOUHSC_02137	sdcS	Sodium-dependent dicarboxylate transporter	-2.34	0.014902
sa_c485s314_at	SAOUHSC_00962		Putative uncharacterized protein	-2.34	0.001246
sa_c7041s6150_a_at	SAOUHSC_00319		Putative uncharacterized protein	-2.34	0.007571
sa_c7422s6445_a_at	SAOUHSC_00438		Alpha amylase family protein, putative	-2.34	0.001506
sa_c4917s4223_a_at	SAOUHSC_00199		Acetate CoA-transferase YdiF	-2.35	0.010221
sa_c2670s2245_a_at	SAOUHSC_01671		Diacylglycerol kinase, putative	-2.37	0.031066
sa_c6021s5217_a_at	SAOUHSC_00226		Putative uncharacterized protein	-2.38	0.008936
sa_c6390s5558_at	SAOUHSC_02925		Putative uncharacterized protein	-2.38	0.027686
sa_c9s10_at	SAOUHSC_00003		Putative uncharacterized protein	-2.38	0.01218
sa_c1078s861_at	SAOUHSC_01142	mraZ	Transcriptional regulator	-2.4	0.005102
sa_c3778s3248_a_at	SAOUHSC_00156		Putative uncharacterized protein	-2.4	0.011886

sa_c3322s2866_a_at	SAOUHSC_01869		Putative uncharacterized protein	-2.41	0.000683
sa_c3414s2943_at	SAOUHSC_01898		Putative uncharacterized protein	-2.41	0.00953
sa_c6010s5210_a_at	SAOUHSC_02812		Putative uncharacterized protein	-2.41	0.000371
sa_c85s79_a_at		orfX	OrfX (Fragment)	-2.41	0.013493
sa_c7311s10178_at	SA0394		Uncharacterized protein	-2.43	0.040007
sa_c6579s5745_a_at	SAOUHSC_02983	gtf2	Glycosyltransferase stabilizing protein	-2.44	0.008217
sa_c9552s10406_a_at	SA0191		Uncharacterized protein	-2.44	0.040853
sa_c592s9345_a_at	SAOUHSC_00994	atl; nag	Bifunctional autolysin	-2.46	0.003138
sa_c9528s8308_a_at	SAOUHSC_00086		3-ketoacyl-acyl carrier protein reductase, putative	-2.46	0.004247
sa_c1088s869_a_at	SAOUHSC_01144	ftsL	Cell division protein FtsL	-2.47	0.022438
sa_c2556s2131_at	SAOUHSC_01629	lipM	Octanoyltransferase	-2.47	0.008971
sa_c6062s5256_a_at	SAOUHSC_02829		Putative NAD(P)H nitroreductase	-2.47	0.002067
sa_i10262dr_x_at	SA1636		Uncharacterized protein	-2.47	0.036775
sa_c3448s2976_a_at	SAOUHSC_01910	pckA	Phosphoenolpyruvate carboxykinase	-2.48	0.002938
sa_c6539s5707_a_at	SAOUHSC_02971		Aureolysin, putative	-2.48	0.00659
sa_c5500s4763_a_at	SAOUHSC_02660		Putative uncharacterized protein	-2.49	0.002343
sa_c5496s4759_a_at	SAOUHSC_02659		Putative uncharacterized protein	-2.5	0.003641
sa_c5504s9245_a_at	SAOUHSC_02661		PTS system sucrose-specific IIBC component, putative	-2.5	0.011424
sa_c5544s4800_a_at	SAOUHSC_02675	nreC	Oxygen regulatory protein	-2.5	0.016078
sa_c5187s4486_a_at	SAOUHSC_02608		Putative uncharacterized protein	-2.51	0.002604
sa_c5548s4804_a_at	SAOUHSC_02676	nreB	Oxygen sensor histidine kinase	-2.52	0.009468
sa_c7665s6679_a_at	SAOUHSC_00535		Uncharacterized epimerase/dehydratase	-2.52	0.007247
sa_c6381s5549_a_at	SAOUHSC_00235		Putative uncharacterized protein	-2.53	0.008763
sa_c2501s2080_a_at	SAOUHSC_01613		2-oxoisovalerate dehydrogenase	-2.54	0.006327
sa_c439s269_a_at	SAOUHSC_00950		Putative uncharacterized protein	-2.54	0.009069
sa_c6547s5715_a_at	SAOUHSC_02973		Putative uncharacterized protein	-2.54	0.000161
sa_c700s509_a_at	SAOUHSC_01019		Putative uncharacterized protein	-2.54	0.000067
sa_c7698s6703_a_at	SAOUHSC_00544	sdrC	Serine-aspartate repeat-containing protein C	-2.55	0.001277
sa_i10200u_x_at	SAOUHSC_00537		Putative uncharacterized protein	-2.55	0.035087

sa_c4134s3487_a_at	SAOUHSC_02257		Putative uncharacterized protein	-2.56	0.002111
sa_c6758s5899_a_at	SAOUHSC_03022		UPF0312 protein	-2.56	0.001865
sa_c2678s2252_a_at	SAOUHSC_01673		Putative uncharacterized protein	-2.57	0.026329
sa_c7965s6952_a_at	SAOUHSC_00643		TagB protein, putative	-2.6	0.018204
sa_c10333s9011_a_at	SAOUHSC_01244		Putative uncharacterized protein	-2.61	0.02838
sa_c2707s2281_a_at	SAOUHSC_01685	hrcA	Heat-inducible transcription repressor	-2.61	0.012155
sa_c9589s8350_at	SAOUHSC_01262	recA	Recombinase A	-2.61	0.000065
sa_c5195s4497_a_at	SAOUHSC_02610	hutG	Formimidoylglutamase	-2.62	0.005135
sa_c6732s5872_a_at	SAOUHSC_03016		Putative uncharacterized protein	-2.62	0.001983
sa_c9676s8432_a_at	SAOUHSC_01646		Glucokinase, putative	-2.62	0.004297
sa_c4589s3921_a_at	SAOUHSC_02404		Putative uncharacterized protein	-2.63	0.000398
sa_c7710s6710_a_at	SAOUHSC_00548		Putative uncharacterized protein	-2.63	0.002925
sa_c6173s5353_a_at	SAOUHSC_02862	clpL	ATP-dependent Clp protease ATP-binding subunit	-2.64	0.010622
sa_c2509s2087_a_at	SAOUHSC_01615		DNA repair protein RecN	-2.66	0.002243
sa_c7036s9080_a_at	SAOUHSC_00318		Putative uncharacterized protein	-2.67	0.014088
sa_c5614s4868_a_at	SAOUHSC_02691		Putative uncharacterized protein	-2.68	0.001658
sa_c2666s2241_a_at	SACOL1625	cdd	Cytidine deaminase	-2.7	0.003532
sa_c7389s6417_a_at	SAOUHSC_00429		MutT/nudix family protein, putative	-2.73	0.006965
sa_c9652s8408_a_at	SAOUHSC_01668	era	GTPase	-2.74	0.002453
sa_i10003u2r_x_at	SAOUHSC_02549		Molybdenum ABC transporter, periplasmic molybdate-binding protein	-2.75	0.042267
sa_i8754ur_x_at	SAOUHSC_01363		Putative uncharacterized protein	-2.75	0.000177
sa_c2258s1957_a_at	SAOUHSC_00107		Putative uncharacterized protein	-2.76	0.002964
sa_c704s512_a_at	SAOUHSC_01021		Putative uncharacterized protein	-2.77	0.032379
sa_c9499s10392_a_at	SAOUHSC_00043		Putative uncharacterized protein	-2.77	0.00116
sa_c7287s6347_a_at	SAOUHSC_00386		Putative uncharacterized protein	-2.78	0.001697
sa_c2497s2076_a_at	SAOUHSC_01612		2-oxoisovalerate dehydrogenase	-2.8	0.004768
sa_c4675s3995_a_at	SAOUHSC_02443		Putative uncharacterized protein	-2.8	0.006852
sa_c5435s4704_a_at	SAOUHSC_00647		Putative uncharacterized protein	-2.8	0.004972
sa_c969s761_a_at	SAOUHSC_01104		Succinate dehydrogenase	-2.8	0.001273

sa_c7393s6421_a_at	SAOUHSC_00430		Putative uncharacterized protein	-2.81	0.009095
sa_c9087s10315_s_at	SAOUHSC_01956		Putative uncharacterized protein	-2.81	0.005649
sa_i10084u_x_at	SAOUHSC_02990	sraP; sasA	Serine-rich adhesin for platelets (Staphylococcus aureus surface protein A)	-2.84	0.008494
sa_i2952u_x_at	SAOUHSC_01899		Putative uncharacterized protein	-2.84	0.003355
sa_c5381s4656_a_at	SAOUHSC_00667		ABC transporter ATP-binding protein, putative	-2.87	0.000454
sa_c5616s4872_a_at	SAOUHSC_02692		Antitoxin	-2.89	0.002561
sa_c6938s6059_a_at	SAOUHSC_00285		Putative uncharacterized protein	-2.9	0.00282
sa_c8190s7169_at	SAOUHSC_00715	saeR	Response regulator SaeR	-2.9	0.003836
sa_i5185ur_x_at	SAOUHSC_02804		Putative uncharacterized protein	-2.9	0.047565
sa_c2346s1974_a_at	SAOUHSC_00114		Capsular polysaccharide biosynthesis protein, putative	-2.91	0.003213
sa_c720s531_a_at	SAOUHSC_01027		Putative uncharacterized protein	-2.91	0.010065
sa_c2658s2233_a_at	SAOUHSC_01667	recO	DNA repair protein	-2.92	0.021975
sa_c2861s2424_a_at	SACOL1682		ATPase, AAA family	-2.92	0.002734
sa_c1378s1153_a_at	SAOUHSC_01242	rimP	Ribosome maturation factor	-2.93	0.017287
sa_c4581s3912_a_at	SAOUHSC_02402		PTS system, mannitol-specific IIa component, putative	-2.94	0.001827
sa_c5807s5048_a_at	SAOUHSC_02753		Membrane protein, putative	-2.94	0.010798
sa_c8111s7096_at	SAOUHSC_00690		Putative uncharacterized protein	-2.94	0.003933
sa_c6403s5575_a_at	SAOUHSC_02930		Putative uncharacterized protein	-2.96	0.009801
sa_c10280s8964_a_at	SAOUHSC_00481		Putative uncharacterized protein	-2.98	0.004134
sa_c18s19_a_at	SAOUHSC_00005	gyrB	DNA gyrase subunit B	-2.98	0.00007
sa_c8196s7173_a_at	SAOUHSC_00717		Putative uncharacterized protein	-3	0.017332
sa_c10267s9119_a_at	SACOL1879		Transposase	-3.01	0.008038
sa_c8308s7288_a_at	SAOUHSC_00757	pepT	Peptidase T	-3.05	0.014233
sa_c6129s5308cs_s_at	SAOUHSC_02849		Pyruvate oxidase, putative	-3.08	0.001299
sa_c8459s7421_a_at	SAOUHSC_00821		Putative uncharacterized protein	-3.08	0.013888
sa_c5652s4904_a_at	SAOUHSC_02706	sbi	Immunoglobulin-binding protein	-3.09	0.001308
sa_c22s9176_a_at	SAOUHSC_00006	gyrA	DNA gyrase subunit A	-3.1	0.002266

sa_c10607s11063_a_at	SACOL0359		Uncharacterized protein	-3.13	0.042962
sa_i10436ur_x_at	SAOUHSC_00825		Putative uncharacterized protein	-3.18	0.021153
sa_c8380s7355_a_at	SAOUHSC_00792		Epimerase family protein	-3.21	0.009146
sa_c8473s7434_a_at	SAOUHSC_00826		Putative uncharacterized protein	-3.23	0.001067
sa_c9634s8390_a_at	SAOUHSC_01684	grpE	Protein GrpE (HSP-70 cofactor)	-3.23	0.00122
sa_c1384s1156_a_at	SAOUHSC_01243	nusA	Transcription termination/antitermination protein	-3.24	0.000406
sa_c5556s4813_a_at	SAOUHSC_02677		Putative uncharacterized protein	-3.25	0.011151
sa_c5608s4864_a_at	SAOUHSC_02690		Putative uncharacterized protein	-3.39	0.000892
sa_c2505s2085_a_at	SAOUHSC_01614		Dihydrolipoyl dehydrogenase	-3.41	0.00184
sa_c3949s9846_at	SAOUHSC_02161		MHC class II analog protein	-3.43	0.001884
sa_c8643s7591_a_at	SAOUHSC_00882		Putative uncharacterized protein	-3.52	0.005775
sa_c6058s5252_a_at	SAOUHSC_02828		Putative uncharacterized protein	-3.56	0.000789
sa_c5331s10015_s_at	SAOUHSC_01948		ABC transporter domain protein	-3.58	0.005708
sa_c4671s3991_at	SAOUHSC_02442		Putative uncharacterized protein	-3.6	0.006425
sa_c5660s4913_a_at	SAOUHSC_02708		Gamma-hemolysin h-gamma-ii subunit, putative	-3.63	0.010476
sa_c9491s8284_a_at	SAOUHSC_00036		Putative uncharacterized protein	-3.63	0.000481
sa_c3059s2614_a_at	SAOUHSC_01794		Glyceraldehyde-3-phosphate dehydrogenase	-3.64	0.005142
sa_c7418s6444_a_at	SAOUHSC_00437		Putative uncharacterized protein	-3.69	0.000299
sa_c9481s10390_x_at	SAV1827	yent1	Enterotoxin	-3.72	0.019356
sa_c1390s1164_a_at	SAOUHSC_01245		Putative uncharacterized protein	-3.8	0.000915
sa_c298s136_a_at	SAOUHSC_02994		Putative uncharacterized protein	-3.83	0.013808
sa_c5431s4700_a_at	SAOUHSC_00842	metN2	Methionine import ATP-binding protein	-3.94	0.000245
sa_c7333s6374_a_at	SAOUHSC_00410		Putative uncharacterized protein	-3.95	0.000464
sa_c5429s4696_a_at	SAOUHSC_02754		ABC transporter, ATP-binding protein, putative	-3.99	0.003891
sa_c1084s865_a_at	SAOUHSC_01143	rsmH; mraW	Ribosomal RNA small subunit methyltransferase H	-4.01	0.000832
sa_c10510s10955cv_s_at	SAOUHSC_02662		PTS system sucrose-specific IIBC component	-4.05	0.001398
sa_c3552s9819_at	SA1641		Uncharacterized protein	-4.05	0.044428
sa_c8681s7630_a_at	SAOUHSC_00898	argH	Argininosuccinate lyase	-4.16	0.03496

sa_c13s15_a_at	SAOUHSC_00004	recF	DNA replication and repair protein	-4.2	0.001342
sa_c4528s3873_a_at	SAOUHSC_02387		Putative uncharacterized protein	-4.29	0.032074
sa_c8512s7471_a_at	SAOUHSC_00843		Putative uncharacterized protein	-4.3	0.001908
sa_c4097s3454_a_at	SAOUHSC_00173	azoR	FMN-dependent NADH-azoreductase	-4.35	0.000758
sa_c6531s5696_a_at	SAOUHSC_02969	arcA	Arginine deiminase	-4.38	0.016651
sa_c5925s10044_at	SAOUHSC_00054		Uncharacterized lipoprotein	-4.41	0.023536
sa_c5668s4921_a_at	SAOUHSC_02710	hlgB	Gamma-hemolysin component B	-4.52	0.009031
sa_i4366d_x_at	SAOUHSC_02572		Putative uncharacterized protein	-4.55	0.030581
sa_c8250s7228_a_at	SAOUHSC_00736		Putative lipid kinase	-4.63	0.004732
sa_c9902s10445_at	SAOUHSC_02188		Phage head-tail adaptor, putative	-4.7	0.033738
sa_c6131s5312_a_at	SAOUHSC_02850	cidB	Holin-like protein	-5.03	0.001963
sa_c130s9468_a_at	SA0042		Uncharacterized protein	-5.14	0.018479
sa_c7499s6519_a_at	SAOUHSC_00468		Putative uncharacterized protein	-5.28	0.000749
sa_c47s43_a_at	SAOUHSC_00013		Homoserine O-acetyltransferase	-5.47	0.003433
sa_i11103dr_x_at	SA1208		Uncharacterized protein	-5.48	0.017694
sa_i4668dr_x_at	SAOUHSC_02002		Putative uncharacterized protein	-5.63	0.030129
sa_i10401ur_x_at	SAOUHSC_00083.1		Putative uncharacterized protein	-5.83	0.045376
sa_c8799s7739_a_at	SAOUHSC_0050<br>0	pdxT	Pyridoxal 5'-phosphate synthase subunit	-5.95	0.000092
sa_c4667s3987_a_at	SAOUHSC_0244<br>1	asp23	Alkaline shock protein 23	-6.12	0.000397
sa_c5664s4917_a_at	SAOUHSC_0270<br>9	hlgC	Gamma-hemolysin component C	-6.61	0.002693
sa_c7895s6886_a_at	SAOUHSC_00619		Putative uncharacterized protein	-6.86	0.002221
sa_i9869dr_x_at	SAOUHSC_02176		Conserved hypothetical phage protein	-6.88	0.022378
sa_c10407s10824_a_at	SAOUHSC_02046		Conserved hypothetical phage protein	-7.33	0.008613
sa_c8434s7406_a_at	SAOUHSC_00812	clfA	Clumping factor A	-8.03	0.00118
sa_c8685s7634_a_at	SAOUHSC_00899	argG	Argininosuccinate synthase	-8.44	0.031617
sa_c8807s7748_a_at	SAOUHSC_00502	ctsR	Transcriptional regulator	-8.61	0.014452
sa_c7428s10194_at	SAOUHSC_00440		Putative uncharacterized protein	-8.86	0.032641

sa_c256s9573_s_at	SAOUHSC_00505	clpC	ATP-dependent Clp protease ATP-binding subunit	-9.02	0.001908
sa_c8795s7735_a_at	SAOUHSC_00499	pdxS	Pyridoxal 5'-phosphate synthase subunit	-9.09	0.000204
sa_c4369s3721_at	SAOUHSC_02328	thiE	Thiamine-phosphate synthase	-9.11	0.016013
sa_c278s123_a_at	SAOUHSC_00912	clpB	Chaperone protein	-9.54	0.00207
sa_c8813s7749_a_at	SAOUHSC_00504	mcsB	Protein-arginine kinase	-11.49	0.003188
sa_c7571s6589_a_at	SAOUHSC_00503	mcsA	Protein-arginine kinase activator protein	-11.77	0.005313
sa_c7784s10209_a_at	SA2012		Uncharacterized protein	-12.26	0.008462
sa_c6244s5421_a_at	SAOUHSC_02881	crtP; crtNb	Diapolycopene oxygenase	-13.08	0.014419
sa_i10696u_x_at	SAOUHSC_00974		Glycosyl transferase, group 1	-25.4	0.043503

Figure S5.

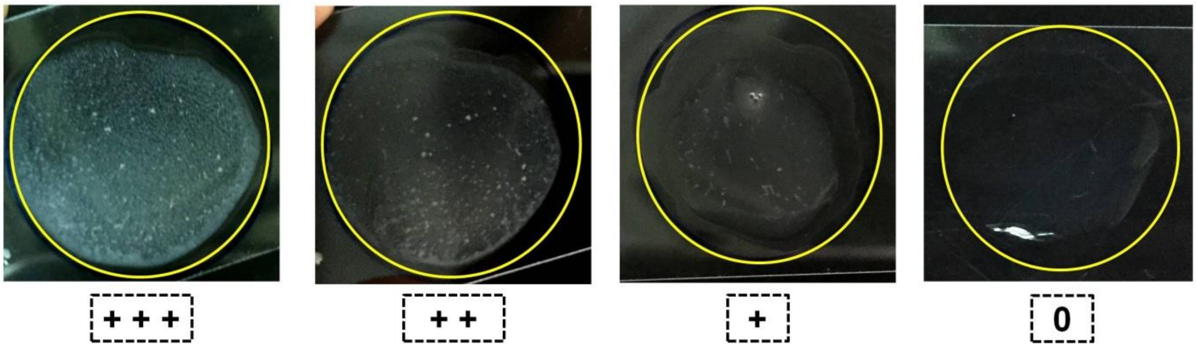

Figure S5. The criteria used for scoring of agglutination/clumps formation of *S. aureus* in the presence and absence of UM-C162. Each tested sample was given a score of +++, ++, + or 0 depending on the macroscopic clumps observed on the slides. A score of 0 represents no clumping activity whilst score +, ++ and +++ indicate the degree of clumps formation in ascending order.

Table S3. Primer sequences used for quantitative RT-PCR analysis.

Gene	Forward sequence (5' to 3')	Reverse sequence (5' to 3')	References
**Housekeeping gene**			
16s rRNA	GGGACCCGCACAAGCGGTGG	GGGTTGCGCTCGTTGCGGGA	[1]
**Upregulated genes**			
SAOUHSC_01311	AAACTTTCTGGTGGTCAACAACG	AGTGCGATGTATAGAGTATCGTTCG	This study
SAOUHSC_01312	TGAATGGCGCCAAAGATTAATGG	ACTGCACTCATTGCAACACCT	This study
SAOUHSC_01626	CTGGCATGACTGGTGCAGAA	ATCGTACGAGCCAGCATTGG	This study
SAOUHSC_01966	GGCAATGGTATCAATCGGGACT	CACAGGCAAAGCAACCATCAC	This study
SAOUHSC_02646	AGGCGTCGTCTTTTTCCTTGG	CCGGCAAGCATTGCTAAAGG	This study
**Downregulated genes**			
saeR	CTGCCAAAACACAAGAACATGATAC	CTTGGACTAAATGGTTTTTTGACATAGT	[2]
clfA	ACCCAGGTTCAGATTCTGGCAGCG	TCGCTGAGTCGGAATCGCTTGCT	[1]
clfB	AACTCCAGGGCCGCCGGTTG	CCTGAGTCGCTGTCTGAGCCTGAG	[1]
eno	TGCCGTAGGTGACGAAGGTGGTT	GCACCGTGTTCGCCTTCGAACT	[1]
gyrB	AGTAACGGATAACGGACGTGGTA	CCAACACCATGTAAACCACCAGAT	[3]
arcA	GTGGTTGACTCATACATCTAGGGC	AGACCAGGCGTTGTAGTGACTTA	[3]
sbi	ATACATCAAAACATTACGCGAACAC	CTGGGTTCTTGCTGTCTTTAAGTG	[2]

References

1 Atshan, S. S. *et al.* Quantitative PCR analysis of genes expressed during biofilm development of methicillin resistant *Staphylococcus aureus* (MRSA). *Infect Genet Evol* **18**, 106-112, doi:10.1016/j.meegid.2013.05.002 (2013).

2 Voyich, J. M. *et al.* The SaeR/S gene regulatory system is essential for innate immune evasion by *Staphylococcus aureus*. *J Infect Dis* **199**, 1698-1706, doi:10.1086/598967 (2009).

3 Beenken, K. E. *et al.* Global gene expression in *Staphylococcus aureus* biofilms. *J Bacteriol* **186**, 4665-4684, doi:10.1128/JB.186.14.4665-4684.2004 (2004).
